# CLE19 suppresses brassinosteroid signaling output via the BSL‐BIN2 module to maintain BES1 activity and pollen exine patterning in *Arabidopsis*

**DOI:** 10.1111/jipb.70024

**Published:** 2025-08-28

**Authors:** Shuangshuang Wang, Shiting Zhang, Ying Yu, Jianzheng Wang, Jingya Wang, Mengyu Li, Jianan Lu, Juanying Ye, Hanji Li, Yeqiao Liu, Yuhan Zhao, Wen Song, Juan Dong, Jia Li, Chunming Liu, Hong Ma, Fang Chang

**Affiliations:** ^1^ State Key Laboratory of Genetics and Development of Complex Phenotypes, School of Life Sciences Fudan University Shanghai 200438 China; ^2^ Department of Biology, the Huck Institutes of the Life Sciences The Pennsylvania State University University Park 16802 PA USA; ^3^ State Key Laboratory of Plant Environmental Resilience, College of Biological Sciences China Agricultural University Beijing 100193 China; ^4^ The Waksman Institute of Microbiology, Rutgers the State University of New Jersey Piscataway 08854 NJ USA; ^5^ Department of Plant Biology Rutgers, the State University of New Jersey New Brunswick 08901 NJ USA; ^6^ Guangdong Provincial Key Laboratory of Plant Adaptation and Molecular Design, School of Life Sciences Guangzhou University Guangzhou 510006 China; ^7^ School of Advanced Agricultural Sciences Peking University Beijing 100871 China; ^8^ Present address: School of Life Sciences East China Normal University Shanghai 200241 China

**Keywords:** *Arabidopsis*, BES1, brassinosteroid signaling, BSLs, CLE19, nuclear export, peptide–hormone crosstalk, phosphoproteomics, pollen exine, tapetum

## Abstract

The pollen exine serves as a protective barrier and signaling interface essential for male fertility in flowering plants. Its precise patterning depends on coordinated interactions between microspores and tapetal cells. While the CLAVATA3/EMBRYO SURROUNDING REGION‐related 19 (CLE19) peptide has been identified as a microspore‐derived “brake” that restricts tapetal activity to maintain exine developmental homeostasis, how CLE19 integrates with hormonal signaling pathways remains poorly understood. Here, we demonstrate that CLE19 attenuates brassinosteroid (BR) signaling output by engaging a defined BSL–BIN2–BES1 signaling cascade. Through quantitative phosphoproteomic analysis, we identified that CLE19 affects the phosphorylation of multiple BR signaling components, including BSL‐type phosphatases BSL1/2/3, the GSK3‐like kinase BIN2, and the transcription factor BES1. We show that CLE19 is perceived by its receptor PXL1, which directly interacts with BSL‐type phosphatases to activate the GSK3‐like kinase BIN2, leading to phosphorylation of BES1 at serine residues S219 and S223. Functional analyses using phospho‐dead and phospho‐mimic BES1 variants confirm that CLE19‐dependent phosphorylation controls BES1 nuclear export and degradation, ultimately suppressing BR‐responsive transcriptional outputs required for pollen exine patterning. Together, our findings define a peptide–hormone signaling axis that regulates transcription factor activity through post‐translational regulation, providing mechanistic insight into how developmental robustness is maintained via intercellular signal integration in plant reproduction.

## Graphical abstract

Antagonistic crosstalk between the microspore‐derived CLE19 peptide and brassinosteroid signaling preserves pollen developmental homeostasis in Arabidopsis. CLE19 activates a protein phosphatase—kinase cascade to phosphorylate the transcription factor BES1, triggering its inactivation and suppressing brassinosteroid signaling outputs, thereby fine tuning male fertility under fluctuating conditions.

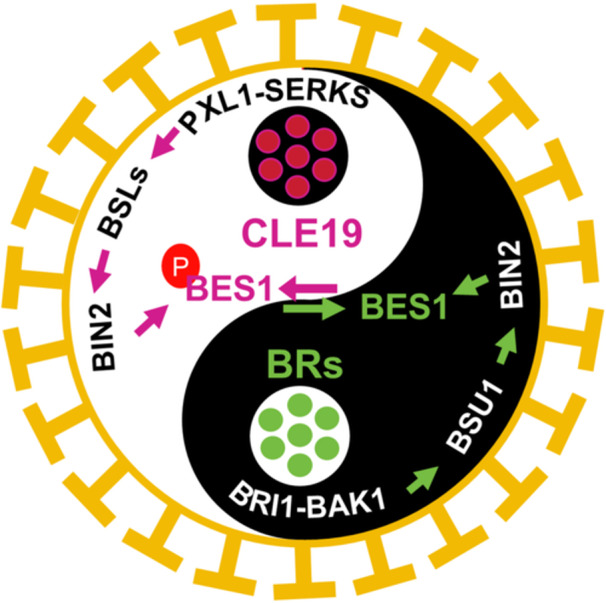

## INTRODUCTION

In flowering plants, the production of viable pollen is essential for sexual reproduction and crop productivity. A key determinant of pollen viability is the formation of the pollen exine, a highly sculpted and chemically resilient outer wall that functions not only as a physical protective barrier but also as a molecular interface for pollen–sigma recognition and environmental protection (Heslop‐Harrison, 1971).

Exine formation is a complex and tightly regulated process involving tapetum‐derived enzymes, lipid precursors, and reciprocal signaling between developing microspores and surrounding tapetal cells (Huang, 2007; Mariani et al., 1990; Stieglitz and Stern, 1973; Piffanelli et al., 1998). Following meiosis, the tapetum facilitates microspore release by degrading the callose wall and supplies sporopollenin precursors required for exine biogenesis. Disruption of this coordination often results in defective exine structure and male sterility, highlighting the importance of precise intercellular regulation.

At the core of this regulation lies a conserved transcriptional cascade involving key transcription factors (TFs) such as *DYSFUNCTIONAL TAPETUM1 (DYT1)*, *bHLH010/089/091*, *MYB35/DEFECTIVE IN TAPETAL DEVELOPMENT AND FUNCTION (TDF1)*, and *ABORTED MICROSPORE (AMS)*, which coordinate tapetum development and sporopollenin biosynthesis. Mutations in these TFs often result in defective pollen exine patterning and male sterility (Zhang et al., 2006; Zhu et al., 2008, 2011., 2015; Ge et al., 2010; Xu et al., 2010, 2014; Chang et al., 2011; Feng et al., 2012; Gómez et al., 2015; Cui et al., 2016).

Among these regulators, DYT1 functions as a molecular “gatekeeper”. Through integrating feedforward and positive feedback loops with downstream TFs, DYT1 amplifies the transcription program required for rapid pollen wall precursor sporopollenin production (Zhu et al., 2015; Cui et al., 2016). To prevent excessive activation, negative regulatory mechanisms such as those mediated by caffeoyl‐CoA O‐methyltransferase 1 (CCoAOMT1) attenuate basic Helix‐Loop‐Helix (bHLH)010/bHLH089 activity through post‐translational modification, promoting their nuclear export and degradation (Lai et al., 2022; Lai et al., 2023).

In addition to transcriptional control, intercellular signaling peptides, particularly members of the CLAVATA3/EMBRYO SURROUNDING REGION‐related (CLE) family, play critical roles in maintaining developmental homeostasis. Among them, CLE19 is produced by developing microspores and acts as a diffusible “brake” signal to prevent excessive activation of tapetal transcriptional programs (Wang et al., 2017). CLE19 is perceived by its receptor‐coreceptor complex composed of PXY‐LIKE1/2 (PXL1/2) and SOMATIC EMBRYOGENESIS RECEPTOR KINASEs (SERKs) on the tapetal cell plasma membrane, initiating intracellular signaling that limits the expression of key TFs such as *AMS* (Wang et al., 2017; Yu et al., 2023). However, the intracellular signaling events that transduce CLE19 signals from the plasma membrane to the nuclear effectors remain poorly characterized.

Brassinosteroids (BRs) are a class of plant steroid hormones that regulate diverse developmental processes including cell elongation, division, and differentiation (Nolan et al., 2020; Li et al., 2025). In reproductive tissues, BR signaling is essential for anther development and pollen viability. Loss‐of‐function mutations in BR receptor BRASSINOSTEROID INSENSITIVE 1 (BRI1) or BR‐responsive TFs BES1 and its homologs often lead to reduced male fertility, highlighting the critical roles of BR signaling in pollen development (Ye et al., 2010; Chen et al., 2019a, 2019b). Canonical BR signaling is transduced through the BRI1–BSL–BIN2–BES1/BZR1 module, in which BR perception by BRI1 at the plasma membrane triggers a phosphorylation cascade that inhibits the GSK3‐like kinase BIN2, thereby allowing dephosphorylated BES1/BZR1 to accumulate in the nucleus and activate downstream gene expression (Clouse et al., 1996; Yin et al., 2002). Although BRs have been implicated to regulate tapetum function and pollen development, whether and how BR signaling is integrated with CLE19 peptide signaling remains unknown.

In this study, we identify a previously uncharacterized mechanistic link between CLE19 and BR signaling. Specifically, we show that CLE19 promotes BES1 phosphorylation and degradation through a BSL–BIN2‐dependent pathway, acting independently of BR biosynthesis or receptor complex formation. Our findings established a dual‐signaling framework through which microspore‐derived peptide cues fine‐tune tapetal hormone‐responsive transcriptional outputs to ensure pollen exine integrity.

## RESULTS

### CLE19‐modulated phosphoproteome reveals enrichment of BR signaling components

**Figure 1:**
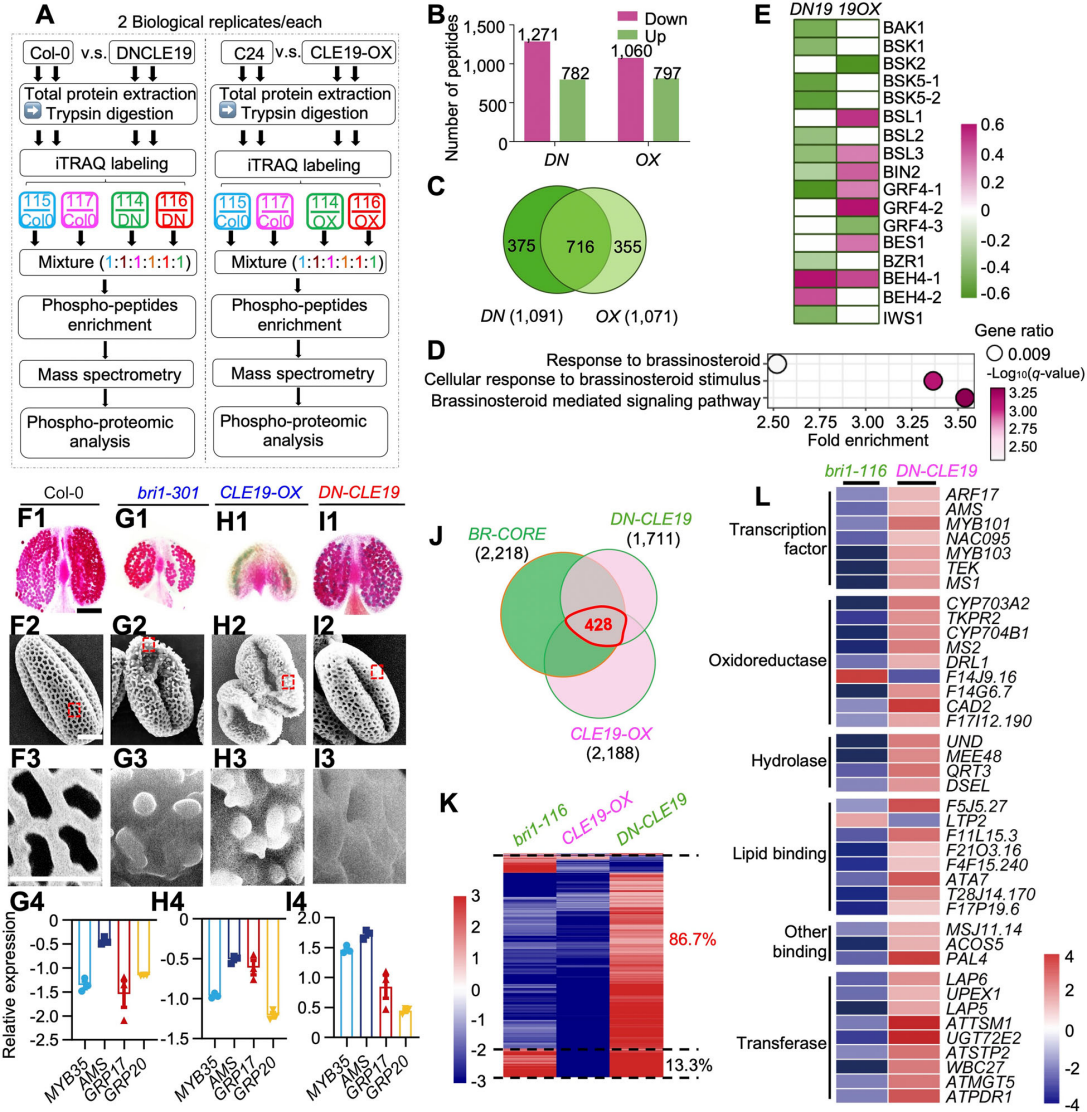
CLE19 modulates the phosphorylation status of BR signaling components and antagonizes BR function in pollen exine formation **(A)** Schematic diagram illustrating the tissue collection and workflow for quantitative phospho‐proteomics using Stage 1–10 *Arabidopsis* floral buds from wild‐type (WT), *CLE19‐OX* (overexpressing), and dominant‐negative (*DN*)*‐CLE19* lines. **(B)** Bar graphs showing the number of differentially phosphorylated peptides in *DN‐CLE19* and *CLE19‐OX*. Pink and green indicated down‐ and up‐regulated peptides relative to WT, respectively. **(C)** Venn diagram showing the overlap of differentially phosphorylated proteins (DPPs) in *DN‐CLE19* and *CLE19‐OX* samples. **(D)** Gene Ontology (GO) enrichment analysis highlighting BR‐related biological processes among CLE19‐regulated phosphoproteins. **(E)** Heatmap showing overlapping proteins with altered phosphorylation in both *DN‐CLE19* and *CLE19‐OX* samples. **(F1**–**I1)** Alexander's stain of anthers from Col‐0 **(F1)**, *bri1‐301* **(G1)**, *CLE19‐OX* **(H1)**, and *DN‐CLE19* **(I1)** plants. **(F2**–**I2)** Scanning electron microscopy (SEM) images of single mature pollen grains showing pollen exine structure. **(F3**–**I3)** Enlarged SEM views highlighting detailed pollen exine morphology of indicated area from **(F2**–**I2)**. **(G4**–**I4)** Quantitative real‐time polymerase chain reaction analysis of pollen exine marker genes (*MYB35*, *AMS*, *GRP17*, *GRP20*) in inflorescences of indicated genotypes. **(F1**–**F3)** Col‐0; **(G1**–**G4)** *bri1‐301*; **(H1**–**H4)** *CLE19‐OX*; **(I1**–**I4)** *DN‐CLE19*. Scale bars: 200 μm **(F1**–**I1)** for pollen grains; 2 μm **(F2**‐**I2)**; 2 μm **(F3**–**I3)**. **(J)** Venn diagram showing the 428 overlapping genes co‐regulated by CLE19 and BR signaling pathways. **(K)** Heatmap showing expression changes of the 428 overlapping genes in *bri1‐116, DN‐CLE19* and *CLE19‐OX*. **(L)** Heatmap showing fold changes of genes involved in tapetum function and pollen wall biosynthesis in *bri1‐116* and *DN‐CLE19* backgrounds.

To understand how the microspore‐derived CLE19 signal regulates tapetal function and pollen exine development, we performed iTRAQ‐based quantitative phosphoproteomics using Stage 1–10 floral buds from *Arabidopsis* wild‐type (WT), *CLE19‐*overexpression (*CLE19‐OX*), and dominant‐negative CLE19 (*DN‐CLE19*) transgenic plants (Figure 1A). Compared to WT, 782 and 1,271 phosphopeptides were up‐ and down‐regulated, respectively, in *DN‐CLE19*, while 797 and 1,060 phosphopeptides were up‐ and down‐regulated, respectively, in *CLE19‐OX* lines (Figure 1B). These phosphopeptides corresponded to 1,091 and 1,071 differentially phosphorylated proteins (DPPs) in *DN‐CLE19* and *CLE19‐OX* backgrounds, respectively, with 716 DPPs shared between the two genotypes (Figure 1B, C; Datasets S1, S2). These results suggest that CLE19 signaling induces broad and specific changes in protein phosphorylation.

Gene Ontology and pathway enrichment analyses revealed a significant overrepresentation of BR signaling components among the DPPs (Figure 1D). Specifically, the phosphorylation level of 13 key BR signaling regulators were altered in *CLE19‐OX* and/or *DN‐CLE19* plants, including BAK1, BSK1/2/5, BSU‐LIKE1/2/3 (BSL1/2/3), BIN2, BZR1, and BES1, as supported by 17 phosphorylated peptides (Figure 1E). Among these, BSK1, BSL1, BIN2, and BES1 showed reduced phosphorylation in *DN‐CLE19* and/or elevated phosphorylation in *CLE19‐OX* plants (Figure 1E), suggesting that CLE19 positively regulates the phosphorylation of BR signaling components. These findings position CLE19 upstream of core BR signaling components, potentially functioning in parallel with or converging upon the canonical BR pathway.

### CLE19 opposes BR signaling to regulate pollen exine formation

To evaluate the functional relationship between CLE19 and BR signaling during pollen development, we compared pollen phenotypes of the BR receptor mutant *bri1‐301*, *CLE19‐OX*, and *DN‐CLE19* transgenic plants. In comparison to WT (Figure 1F1–F3), *bri1‐301* mutant produced smaller anthers and defective pollen exine with weakly connected exine structures (Figure 1G1–G3), resembling *CLE19‐OX* phenotypes (Figure 1H1–H3) while opposite to the excessively thickened and overly structured exine observed in *DN‐CLE19* (Figure 1I1–I3). Quantitative real‐time polymerase chain reaction (RT‐PCR) analysis revealed that transcription levels of four key pollen exine‐related marker genes, *MYB35*, *AMS*, *GRP17*, and *GRP20*, were significantly decreased in both *CLE19‐OX* and *bri1‐301* floral buds, whereas they were dramatically increased in *DN‐CLE19* buds (Figure 1G4, H4, I4). These results support that CLE19 suppresses BR‐mediated promotion of pollen exine development.

To substantiate this opposing relationship at the transcriptomic level, we performed a comparative analysis between CLE19‐ and BR‐responsive gene networks. A total of 923 genes were consistently differentially expressed in both *DN‐CLE19* and *CLE19‐OX* lines (Wang et al., 2017), representing the core CLE19‐responsive gene set (CORE‐CLE) (Figure S1A). Similarly, 2,218 genes were consistently affected in both *cpd* and *bri1‐116* mutants (Ye et al., 2010), forming the core BR‐responsive datasets (CORE‐BR) (Figure S1B). Cross‐comparison revealed 428 overlapping genes (Figure 1J; Datasets S3), with 86.7% (371 genes) exhibiting opposite regulation pattern: 337 genes were up‐regulated in *DN‐*CLE19 and down‐regulated in *bri1‐116*, while 34 exhibited the reverse trend (Figure 1K).

Gene Ontology analysis of these 371 antagonistically regulated genes showed significant enrichment in reproductive development processes such as pollen wall assembly, pollen exine formation, morphogenesis‐related cellular component assembly (Figure S2). Importantly, many key regulators of anther and pollen developmental, such as *ARF17*, *AMS*, *MYB101*, *MYB103*, *MS1*, *CYP703A2, CYP704B1, MALE STERILITY2* (*MS2*), *UNDEAD* (*UND*), *MATERNAL EFFECT EMBRYO ARREST48* (*MEE48*), *QUARTET3* (*QRT3*), *LESS ADHESIVE POLLEN5 (LAP5)*, *LAP6*, *WBC27*, and *ARABIDOPSIS THALIANA ANTHER7* (*ATA7*), were consistently up‐regulated in *DN‐CLE19* but down‐regulated in *bri1‐116* (Figure 1L). These results support a model in which CLE19 and BR signaling operate in opposition to fine‐tune transcriptional programs underlying pollen exine development.

### CLE19 does not alter BR biosynthesis or interfere with the BRI1 receptor complex

Given the observed suppression of BR signaling by CLE19 and its impact on downstream BR pathway components, we next examined whether CLE19 acts upstream, either by altering BR biosynthesis or by interfering with the BR receptor complex.

To evaluate the effect of CLE19 on BR biosynthetic gene expression, we compared CLE19‐regulated transcriptomes with BR signaling pathways. While BR‐mediated signaling pathways were significantly enriched in the CLE19‐affected phosphoproteome (Figure 1D), no such enrichment was detected in the CLE19‐regulated transcriptome (Figure S3A). Quantitative RT‐PCR analysis further confirmed that the transcript levels of key BR biosynthetic genes remained unchanged in *CLE19‐OX* and *DN‐CLE19* plants compared to WT (Figure S3B, C). These results suggest that CLE19 does not modulate BR signaling through transcriptional regulation of BR biosynthesis or signaling genes.

**Figure 2:**
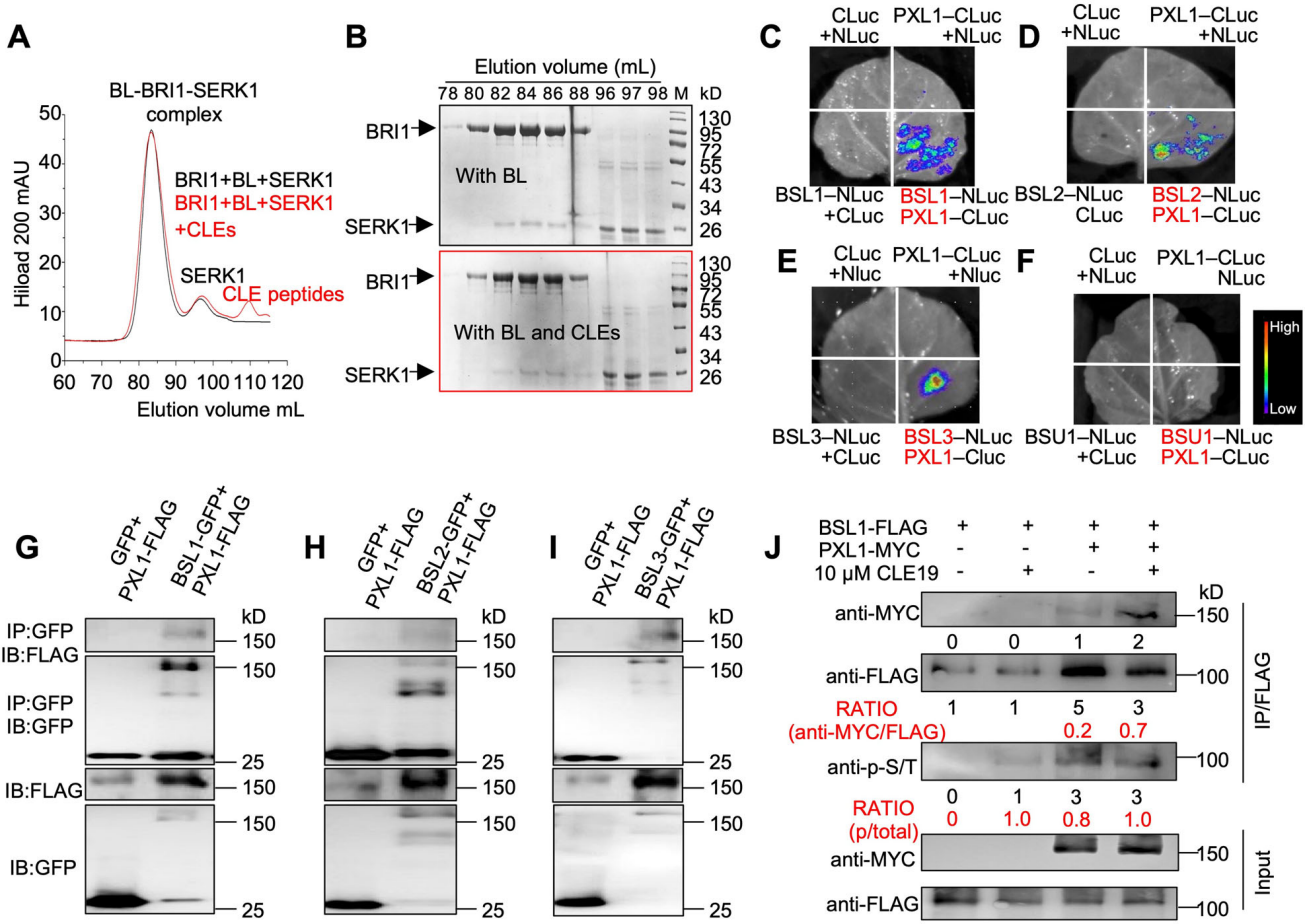
CLE19 does not interfere with BR perception by the BR–BRI1–BAK1 receptor complex and instead signals through direct interaction with BSL phosphatases **(A)** Gel filtration analysis of the BR receptor complex assembly. The addition of various CLE peptides did not disrupt the formation of the BRI1–BL–SERK1 complex, indicating that CLEs do not interfere with BR perception at the receptor level. **(B)** Sodium dodecyl sulfate – polyacrylamide gel electrophoresis (SDS‐PAGE) of fractions from gel filtration experiments in **(A)**, showing the BRI–BL–SERK1 complex elutes at the same position with (the top gel) or without (the bottom gel) CLEs. **(C**–**F)** Luciferase complementation imaging (LCI) assays in *Nicotiana benthamiana* showing that the CLE19 receptor PXL1 physically interacts with BSL1, BSL2, and BSL3, but not with BSU1. **(G**–**I)** Co‐immunoprecipitation (Co‐IP) assays confirming the interactions between PXL1 and BSL1/2/3 *in vivo*. PXL1‐FLAG and BSL1/2/3‐GFP (green fluorescent protein) (or GFP control) were co‐expressed in *Nicotiana benthamiana*. Total protein was extracted and immunoprecipitated using anti‐GFP beads. Western blotting was performed using anti‐FLAG and anti‐GFP antibodies to detect input and immunoprecipitated proteins. **(J)** CLE19 enhances the phosphorylation of BSL1 in a PXL1‐dependent manner. PXL1‐MYC and BSL1‐FLAG were co‐expressed in *Nicotiana benthamiana* with or without 20 μmol/L CLE19 treatment. Total protein was immunoprecipitated using anti‐FLAG beads and analyzed by western blotting with anti‐MYC and anti‐FLAG antibodies, anti‐p‐S/T was used to detect the phosphorylation status of BSL1 protein.

To determine whether CLE19 interferes with the initiation of BR signaling, we tested whether CLE19 affects the formation of the brassinolide (BL)–BRI1–SERK1 receptor complex. Gel filtration assays using recombinant proteins confirmed that BL promotes the formation of a stable BRI1^LRR^‐SERK1^LRR^ complex at pH 4.0, as shown by co‐elution during gel filtration chromatography (Figure 2A, B). If CLE19 or related peptides disrupted the BL–BRI1–SERK1 interaction, we would expect to see a shifts in elution profiles or loss of BRI1–BL–SERK1 complex formation. However, when CLE19 and its functionally redundant family members (CLE9, CLE16, CLE17, CLE41, CLE42, and CLE45) were simultaneously incubated with the mixture containing BL, BRI1^LRR^, and SERK1^LRR^, we observed no disruption in complex formation. The BRI1–BL–SERK1 protein complex eluted consistently at the same position regardless of CLE peptides presence (Figure 2A), and sodium dodecyl sulfate – polyacrylamide gel electrophoresis (SDS‐PAGE) analysis showed no direct CLE–BRI1 interactions (Figure 2B). These findings demonstrate that CLE19 neither competes with BL for BRI1 binding nor interferes with BRI1‐BL‐SERK1 complex assembly.

Together, these findings rule out BR biosynthesis and receptor complex formation as sites of CLE19 action and suggested that CLE19 likely modulates BR signaling at a post‐receptor level. We therefore turned our attention to the CLE19 receptor, PXL1, and its potential interaction with intracellular BR signaling components.

### CLE19 receptor PXL1 interacts with BSL1/2/3

To explore potential downstream partners of the CLE19 receptor PXL1, we performed co‐immunoprecipitation followed by mass spectrometry (Co‐IP/MS) analysis using inflorescence from *PXL1:PXL1‐FLAG* transgenic plants. This analysis identified BSL1, BSL2, and BSL3 as direct interactors of PXL1, representing the first evidence directly connecting CLE peptide signaling to BR‐regulatory phospho‐modulators. These interactions were validated by luciferase complementation imaging (LCI) and Co‐IP assays in a *Nicotiana benthamiana* transient expression system. Strong interactions were consistently observed between PXL1 and BSL1/BSL2/BSL3 in the LCI results, but not with the related phosphatase BSU1 (Figure 2C–F). Co‐immunoprecipitation results further confirmed these direct interactions between PXL1 and BSL1/2/3 (Figure 2G–I).

Given that PXL1 mediates CLE19 signaling during pollen exine formation (Yu et al., 2023), we next tested whether CLE19 peptide enhances the interaction between PXL1 and BSL1 and/or enhances PXL1‐promoted BSL1 phosphorylation. Co‐expression of PXL1–MYC and BSL1–FLAG in *Nicotiana benthamiana*, followed by CLE19 peptide treatment, significantly enhanced both the physical interaction between PXL1 and BSL1 and the phosphorylation level of BSL1 (Figure 2J). These results confirmed that the CLE19 signaling through PXL1 engaged the BSLs–BIN2–BES1 cascade, providing a mechanistic link between extracellular peptide cues and intracellular BR signaling modulation.

### The BSLs–BIN2–BES1 module is expressed in the tapetum and required for pollen exine formation

If CLE19‐PXL1 signaling indeed acts through the BSL–BIN2–BES1 cascade, these components should be expressed in the tapetum and functionally required for normal pollen development. To test this hypothesis, we examined their expression patterns and analyzed pollen phenotypes in corresponding mutant and overexpression lines.

**Figure 3:**
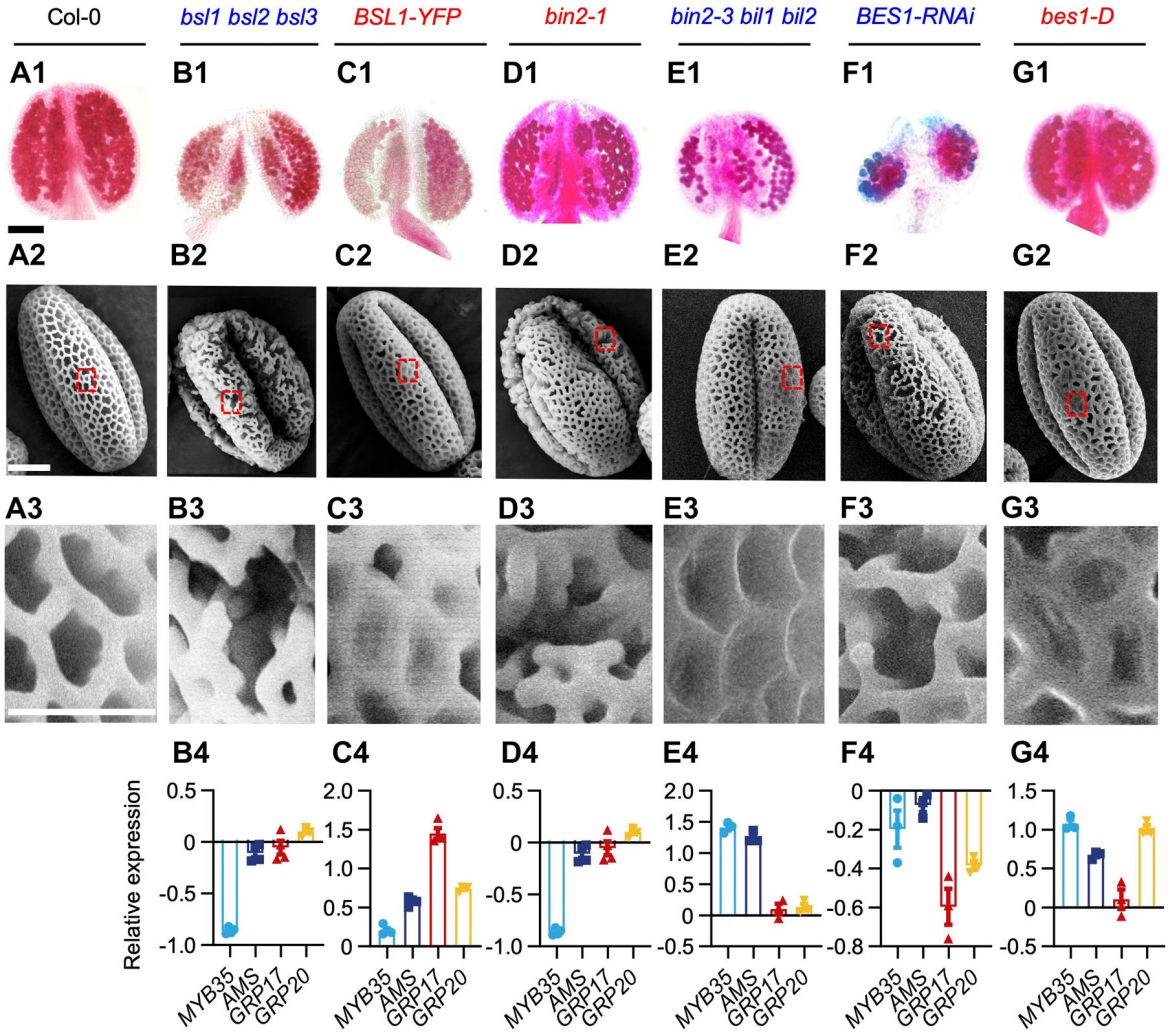
BSLs–BIN2–BES1 cascade is required for pollen exine development **(A1**–**G1)** Alexander staining of anthers from Col‐0, *bsl1 bsl2 bsl3* triple mutants, *BSL1‐YFP* overexpression line, *bin2‐1*, *bin2‐3 bil1 bil2* triple mutants, *BES1*‐RNAi, and *bes1‐D* plants, showing variations in male fertility and pollen viability. **(A2**–**G2)** Scanning electron microscopy (SEM) images of individual mature pollen grains reveal structural differences in exine patterning among genotypes. **(A3**–**G3)** Enlarged SEM images highlight detailed exine morphology from the boxed regions in **(A2**–**G2)**. **(B4**–**G4)** Quantitative real‐time polymerase chain reaction (RT‐PCR) analysis of key pollen exine regulatory genes (*MYB35*, *AMS*, *GRP17*, and *GRP20*) in floral tissues of indicated genotypes. **(A1**–**A3)** Col‐0; **(B1**–**B4)** *bsl1 bsl2 bsl3*; **(C1**–**C4)** *BSL1‐YFP*; **(D1**–**D4)** *bin2‐1*; **(E1**–**E4)** *bin2‐3 bil1 bil2*; **(F1**–**F4)** *BES1‐RNAi*; **(G1**–**G4)** *bes1‐D*. Scale bars: 200 μm in **(A1**–**G1)** for pollen grains; 2 μm in **(A2–G2)** and **(A3–G3)**.

RNA *in situ* hybridization revealed that *BSL1*, *BSL2*, *BSL3*, *BIN2*, and *BES1* are preferentially expressed in the tapetal layer of Stage 6–8 anthers (Figure S4). We further examined pollen exine morphology in both loss‐of‐function mutants (*bsl1 bsl2 bsl3*, *bin2‐3 bil1 bil2*, and *BES1‐RNAi*) and gain‐of‐function lines (*BSL1‐overexpression/OX*, *bin2‐1*, and *bes1‐D*) of BSLs‐BIN2‐BES1 cascade members. Interestingly, *bsl1 bsl2 bsl3*, *bin2‐1*, and *BES1‐RNAi* plants all exhibited fragmented and defective pollen exine structures, phenocopying the *CLE19‐OX* phenotype (Figure 3). In contrast, *BSL1‐OX*, *bin2‐3 bil1 bil2*, and *bes1‐D* produced pollen with excessively thickened exine layers, resembling *DN‐CLE19* plants (Figure 3).

To evaluate molecular changes downstream of these mutations, we perform qRT‐PCR to assess the expression of key pollen exine biosynthesis regulators. Transcription levels of *MYB35, AMS*, *GRP17*, and *GRP20* were significantly reduced in *bsl1 bsl2 bsl3*, *bin2‐1*, and *BES1‐RNAi* lines, compared to WT. In contrast, these genes were up‐regulated in *BSL1‐OX, bin2‐3 bil1 bil2*, and *bes1‐D* plants. Together, these findings collectively support that the BSLs‐BIN2‐BES1 module is not only expressed in the tapetum but also functionally required to regulate the genetic program for pollen exine formation, supporting its role downstream of CLE19–PXL1 signaling.

### CLE19 promotes BES1 phosphorylation via the BSL‐BIN2 cascade

To determine whether the BSL–BIN2–BES1 module functions as a downstream signaling axis of CLE19, we first examined whether CLE19 truly enhances BES1 phosphorylation, and then tested whether this effect depends on BSLs and BIN2.

**Figure 4:**
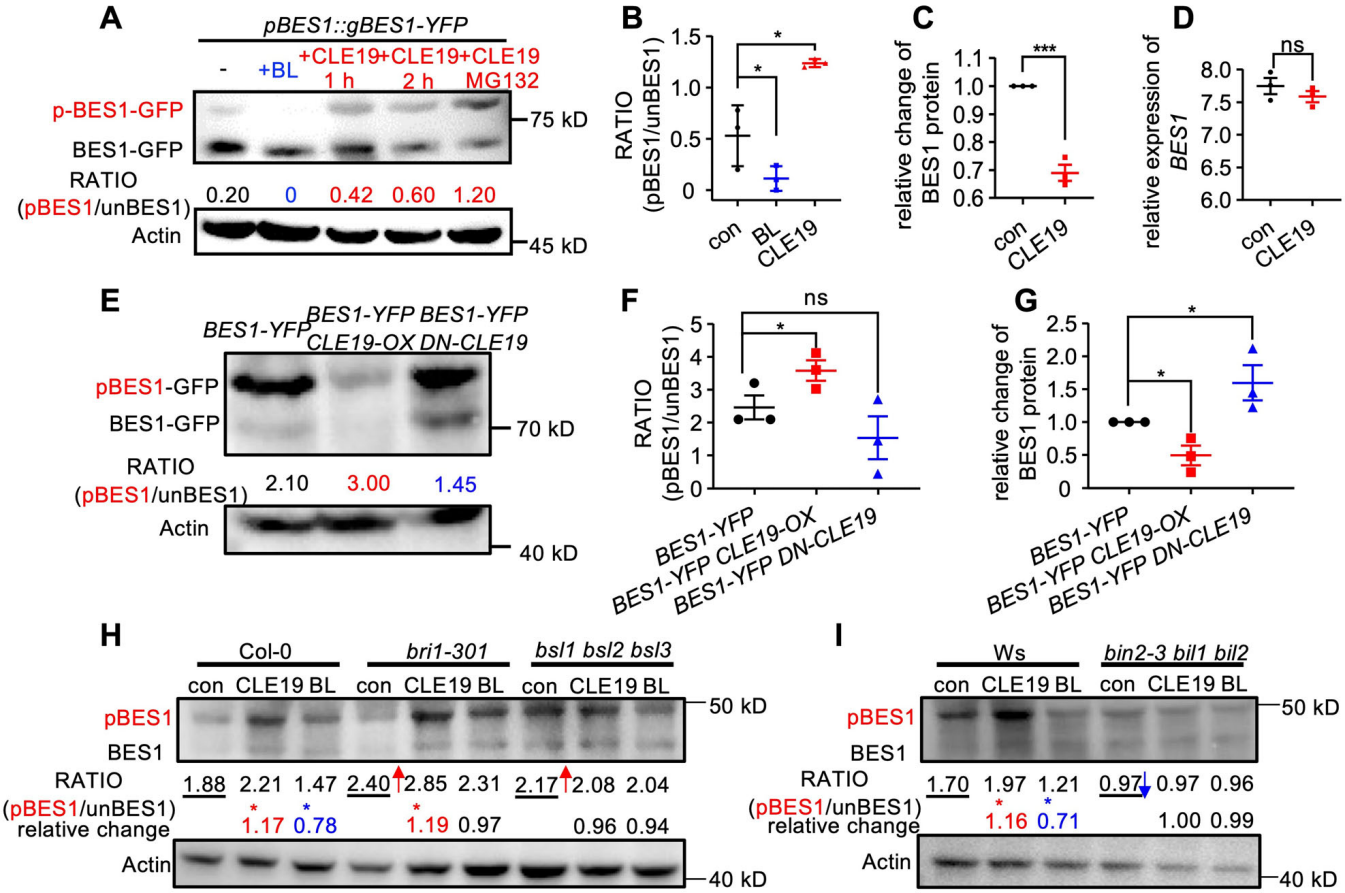
CLE19 promotes BES1 phosphorylation through the BSLs–BIN2 signaling cascade **(A)** Immunoblot analysis showing the phosphorylation status of BES1 in floral buds of *pBES1::gBES1‐YFP* transgenic plants treated with 1/2 Murashige and Skoog (MS) medium (control), 2‐h brassinolide (BL), 1‐h or 2‐h CLE19, or 2‐h CLE19 combined with MG132. **(B)** Quantification of the phosphorylated BES1‐YFP (yellow fluorescent protein) (pBES1) to unphosphorylated BES1‐YFP (unBES1) ratio (pBES1/unBES1) in 1/2 MS control, BL, and CLE19 + MG132. **(C)** Relative change in total BES1 protein levels in *pBES1::gBES1‐YFP* floral buds with or without CLE19 treatment. **(D)** Quantitative real‐time polymerase chain reaction (qRT‐PCR) analysis of *BES1* transcript levels in *pBES1::gBES1‐YFP* floral buds with or without CLE19 treatment. **(E)** Western blot showing BES1 phosphorylation status in wild‐type (WT), *p35S::BES1‐EYFP*, *p35S::BES1‐EYFP CLE19‐OX* and *p35S::BES1‐EYFP DN‐CLE19* seedlings. **(F)** Quantification of the pBES1/unBES1 ratio across the indicated genotypes. **(G)** Relative change in total BES1 protein levels among the indicated genotypes. **(H**, **I)** Immunoblot analysis of BES1 phosphorylation in Col‐0, *bri1‐301*, *bsll bsl2 bsl3* **(H)** and Ws, *bin2‐3 bill bil2* **(I)** seedlings grown on 1/2 MS medium and treated with 2 h with mock (1/2 MS), BL, and CLE19. For each genotype, the basal BES1 phosphorylation level (ratio of pBES1/unBES1) is underlined. Red ↑ and blue ↓ arrows mark increased or reduced basal BES1 phosphorylation level (control, con) relative to the corresponding WT. Treatment‐induced relative change of BES1 phosphorylation were quantified as ((pBES1/unBES1) under treatment) / ((pBES1/unBES1)_in control). Red asterisk (*) indicate obvious increase and blue asterisk (*) indicate obvious decrease of BES1 phosphorylation level compared to mock.

First, CLE19‐induced BES1 phosphorylation was validated by CLE19 peptide treatment assays and co‐expression experiments with BES1 (Figure 4). In *pBES1::gBES1‐YFP* transgenic plants, western blotting analysis revealed two distinct BES1 bands: a slower‐migrating phosphorylated BES1 and a faster‐migrating unphosphorylated BES1 (Figure 4A). Under control conditions, the ratio of phosphorylated‐to‐unphosphorylated BES1 was approximately 0.20. As expected, treatment with BL decreased BES1 phosphorylation. CLE19 treatment significantly increased this ratio to 0.42 and 0.60 after 1‐ and 2‐h, respectively. Notably, co‐treatment with CLE19 and the proteasome inhibitor MG132 further elevated the phosphorylation ratio to 1.2 (Figure 4A, B), suggesting that CLE19‐induced BES1 phosphorylation is coupled with proteasomal degradation. Meanwhile, qRT‐PCR analysis showed no significant changes of *BES1* transcript levels with or without CLE19 treatment (Figure 4C, D), consistent with our transcriptomic data indicating no obvious changes in *BES1* expression in *CLE19‐OX* and *DN‐CLE19* anthers compared to WT anthers (Figure S3D). These results confirmed that CLE19 promotes BES1 degradation at the protein level rather than through transcriptional regulation. Consistently, *BES1‐YFP CLE19‐OX* double transgenic plants showed a significant increase in phosphorylated BES1 levels, accompanied by an obvious reduction in total BES1 protein compared to *BES1‐YFP* alone. In contrast, no such increase was observed in *BES1‐YFP DN‐CLE19* plants (Figure 4E–G). Together, these findings provide strong evidence that CLE19 positively regulates BES1 phosphorylation and promotes its degradation through the proteasome pathway.

To determine whether CLE19‐induced BES1 phosphorylation is mediated through the BSL–BIN2 signaling cascade, we treated 10‐d‐old seedlings of WT (Col‐0 and Ws), *bri1301 bsl1 bsl2 bsl3*, and *bin2‐3 bil1 bil2* mutants with CLE19 peptide, using BL as a reference control. Consistent with our observation in floral buds, CLE19 treatment increased BES1 phosphorylation in both Col‐0 and Ws seedlings, whereas BL treatment reduced it (Figure 4H, I). In *bri1‐301* and *bsl1 bsl2 bsl3* mutants, the basal level of BES1 phosphorylation was significantly elevated compared to the WT (Figure 4H, I). This is consistent with known BR signaling mechanisms: BRI1 deficiency impairs BL perception, while loss of BSL phosphatases relieves inhibition on BIN2 kinase, both resulting in the accumulation of phosphorylated BES1 due to reduced dephosphorylation. By contrast, the *bin2‐3 bil1 bil2* triple mutant exhibited obviously reduced BES1 phosphorylation compared to the Ws control (Figure 4I), supporting the role of BIN2/BIL2/BIL3 as positive regulators of BES1 phosphorylation. Importantly, BL treatment had minimal or no effect on BES1 phosphorylation in *bri1‐301*, *bsl1 bsl2 bsl3*, or *bin2‐3 bil1 bil2* mutants (Figure 4H, I), confirming that BL‐mediated BES1 dephosphorylation depends on these core components. In contrast, CLE19 treatment significantly increased BES1 phosphorylation in *bri1‐301*, but not in *bsl1 bsl2 bsl3* or *bin2‐3 bil1 bil2* mutants (Figure 4H, I). This differential response indicates that CLE19‐mediated BES1 phosphorylation is independent of BRI1, but strictly depends on functional BSL1/2/3 and BIN2‐related kinases.

### CLE19 induces BES1 nucleus export and degradation

**Figure 5:**
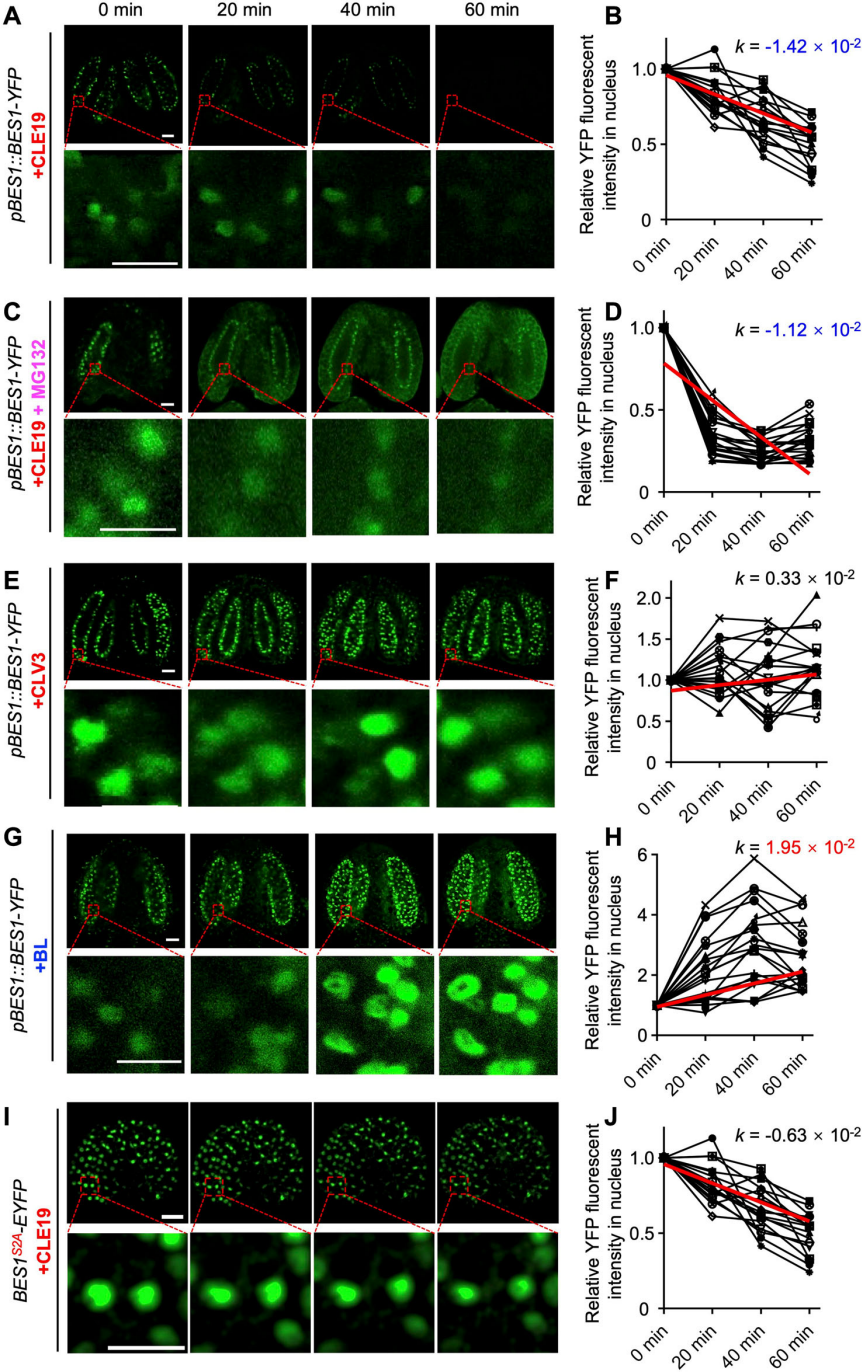
CLE19 induces BES1 nuclear export and degradation **(A**–**H)** Confocal microscopy images of *pBES1::BES1‐YFP* anthers and relative yellow fluorescent protein (YFP) fluorescence intensity in tapetal cell nucleus under the treatment of CLE19 **(A**, **B)**, CLE19 plus MG132 **(C**, **D)**, CLV3 **(E**, **F)**, brassinolide (BL) **(G**, **H)**, and images of *35S::BES1* ^*S2A*^ *‐EYFP* anther treated with CLE19 **(I**, **J)**. Sequential confocal imaging was performed on the same anther, and fluorescence intensity of nuclear‐localized BES1‐YFP in individual tapetal cells was quantified over time. To minimize variability and ensure internal consistency, we normalized the fluorescence intensity of each nucleus to its initial time‐point value (set as 1), and plotted the relative changes at subsequent time points. Each line in (**B**, **D**, **F**, **H**, **J**) represents the nuclear fluorescence variation of an independent tapetal cell, and 20 independent cells are quantified in each treatment (*SD*). A larger absolute value of the slope (*k*) indicates a more significant change in nuclear fluorescence signal. Scale bars: 25 μm (upper of **A**, **C**, **E**, **G**, **I**), 50 μm (lower of **A**, **C**, **E**, **G**, **I**).

Previous studies have linked BES1 phosphorylation to its nuclear export and degradation (Yin et al., 2002). To investigate whether CLE19 affects the subcellular localization of BES1, we treated *pBES1::gBES1‐YFP* transgenic plants with synthetic CLE19 peptide. In untreated tapetal cells, BES1‐YFP fluorescence was predominantly nuclear with weak cytoplasmic signals. However, a 1‐h CLE19 treatment significantly reduced BES1‐YFP fluorescence in both nuclear and cytoplasmic compartments, suggesting that CLE19 promotes BES1 degradation (Figure 5A, B). To decouple nuclear export from degradation, we co‐treated *pBES1::BES1‐YFP* plants with CLE19 and MG132. This combination not only increased the ratio of phosphorylated‐to‐unphosphorylated BES1 proteins, but also caused a pronounced accumulation of BES1‐YFP in the cytoplasm while there was a reduction in the nucleus (Figure 5C, D). In contrast, treatment with CLV3, a CLE19 homolog that functions in shoot apical maintenance but not in pollen development, did not affect nuclear BES1 levels (Figure 5E, F). As a control, BL treatment led to enhanced nuclear accumulation of BES1‐YFP, as indicated by a higher fluorescence intensity in the nucleus. (Figure 5G, H). Together, these results demonstrate that CLE19 specifically enhances BES1 phosphorylation, cytoplasm retention, and proteasomal degradation. This reveals a novel mechanism by which peptide signal suppresses BR outputs via regulated TF turnover.

### The S219 and S223 sites are essential for CLE19‐induced BES1 translocation

Previous studies have reported two distinct *BES1* isoforms: the shorter *BES1‐S* (Yin et al., 2002) and the longer *BES1‐L* (Jiang et al., 2015). However, which isoform is functionally relevant in pollen development remains unclear. Our RNA sequencing (RNA‐seq) analysis of Stage 4–10 *Arabidopsis* anthers revealed that *BES1‐S* is the predominantly expressed isoform (Figure S5). Based on these findings, we focused our subsequent analysis on BES1‐S. Notably, the two CLE19‐responsive phosphorylation sites identified in the phosphopeptide QSMTSLNYPFYAV**S**APA**S**PTHHR were located at serine residues 219 and 223 (S219 and S223), situated within the C‐terminal BIN2 phosphorylation domain of BES1 (Figure S6A).

Although previous studies have associated BES1 phosphorylation with its reduced nuclear accumulation, phosphorylation at S219 and S223 has not been previously reported. To investigate the functional significance of these residues, we generated phospho‐deficient BES1 mutants (BES1^S2A^, with S219A/S223A substitutions) fused to enhanced yellow fluorescent protein (EYFP) under the control of the 35S promoter (*35S::BES1* ^*S2A*^ *‐EYFP*), with *35S::BES1‐EYFP* serving as a control. Compared to WT BES1‐YFP, BES1^S2A^ *‐*EYFP showed significantly enhanced nuclear localization in tapetal cells, as quantified by the nucleus‐to‐total fluorescence intensity ratio (Figure S6B, C).

We further tested whether CLE19 regulates BES1 nuclear export through these specific sites. In contrast to CLE19 significantly reducing nuclear accumulation of BES1‐YFP (Figure 5A, B), CLE19 failed to alter the nuclear localization of BES1^S2A^‐EYFP (Figure 5I, J), indicating that S219 and S223 are essential for CLE19‐induced nuclear export of BES1. Consistently, CLE19 treatment did not significantly affect the phosphorylation of BES1^S2A^‐EYFP (Figure S6D). Similarly, BL treatment decreased BES1 phosphorylation in *BES1‐YFP* plants but failed to effect either BES1^S2A^‐EYFP or BES1^S2D^‐EYFP (Figure S6E), suggesting these sites are required for both CLE19‐ and BR‐mediated post‐translational regulation of BES1.

### The S219 and S223 sites are essential for CLE19‐regulated pollen exine development

**Figure 6:**
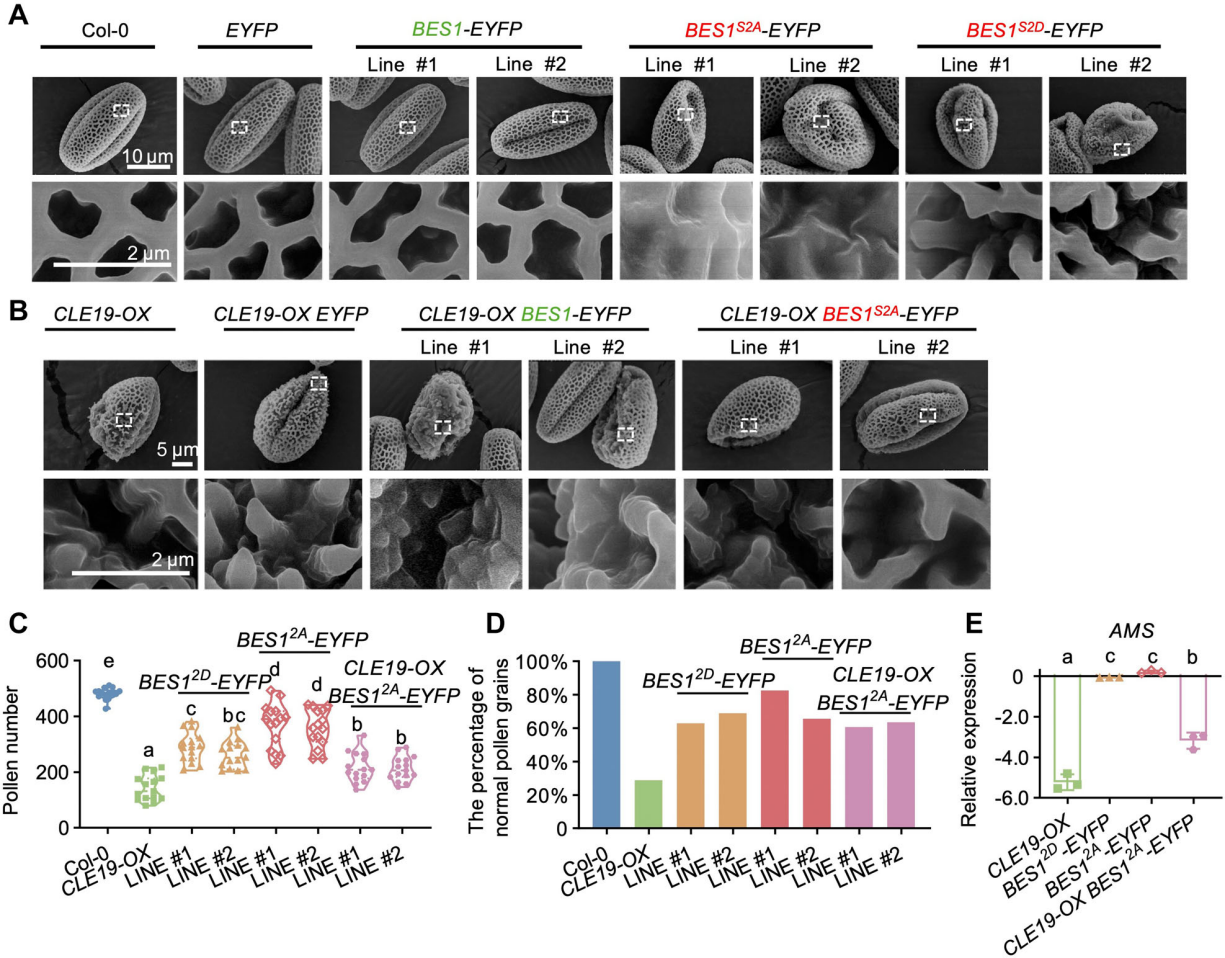
Phosphorylation at S219 and S223 is essential for BES1 function in pollen exine formation **(A)** Scanning electron micrographs of pollen exine from Col‐0, *35S::EYFP*, *35S::BES1‐EYFP*, *35S::BES1* ^*S2A*^ *‐EYFP*, and *35S::BES1* ^*S2D*^ *‐EYFP* transgenic lines. **(B)** Scanning electron microscopy (SEM) images of pollen exine from *CLE19‐OX, CLE19‐OX 35S::EYFP*, *CLE19‐OX 35S::BES1‐EYFP* and *CLE19‐OX 35S::BES1* ^*S2A*^ *‐EYFP* transgenic lines. **(C)** Quantification of pollen grain numbers per anther in each genotype. **(D)** Percentage of pollen grains exhibiting normal exine morphology in each genotype. **(E)** Quantitative real‐time polymerase chain reaction (qRT‐PCR) analysis showing the expression changes of the downstream *AMS* gene in inflorescences of *CLE19‐OX, 35S::BES1* ^*S2A*^ *‐EYFP*, *35S::BES1* ^*S2D*^ *‐EYFP*, and *CLE19‐OX 35S::BES1* ^*S2A*^ *‐EYFP* transgenic lines, relative to the wild‐type. Different letters indicate significant differences (*P* < 0.05). Statistical significance was determined using one‐way analysis of variance (ANOVA) followed by Tukey's multiple comparison test.

To further investigate the functional significance of BES1 phosphorylation at S219 and S223 in pollen development, we examined pollen development in transgenic *Arabidopsis* plants expressing *35S::EYFP*, *35S::BES1‐EYFP*, *35S::BES1* ^*S2A*^ *‐EYFP* (phospho‐dead), and *35S::BES1* ^*S2D*^ *‐EYFP* (phospho‐mimic), alongside WT controls. Plants overexpressing EYFP or BES1‐EYFP showed normal pollen numbers and intact pollen wall structures. In contrast, *BES1* ^*S2A*^ *‐EYFP* plants featured small lacunae filled with excess sporopollenin, phenocopying *DN‐CLE19* phenotypes (Figure 6A). *BES1* ^*S2D*^ *‐EYFP* lines developed pollen with reduced counts and poorly connected exine structures, resembling *CLE19‐OX* phenotypes (Figure 6A).

To assess whether BES1 phosphorylation at S219/S223 sites mediates CLE19‐dependent signaling, we introduced *35S::BES1‐EYFP* and *35S::BES1* ^*S2A*^ *‐EYFP* constructs into the *CLE19‐OX* plants. If BES1 functions downstream of CLE19 and its phosphorylation at S219/S223 is essential for transducing the CLE19 signal, *BES1* ^*S2A*^ overexpression is expected to suppress the *CLE19‐OX* phenotype. As expected, *BES1* ^*S2A*^ *‐EYFP* notably rescued both pollen number and exine patterning in *CLE19‐OX* plants (Figure 6B–D). In addition, qRT‐PCR analysis revealed that expression of CLE19 pathway target genes such as *AMS* was restored in *CLE19‐OX* plants expressing *BES1* ^*S2A*^ *‐EYFP* anthers (Figure 6E). These results demonstrate that BES1 phosphorylation at S219 and S223 serves as a critical molecular switch for CLE19‐mediated repression of BR signaling and downstream control of pollen exine development.

## DISCUSSION

The precise coordination of intercellular signaling is fundamental to plant development and reproductive success. A primary example is the formation of the pollen exine, a highly ornamented outer wall critical for pollen viability, which depends on tightly regulated communications between developing microspores and the surrounding tapetum. While both peptide hormones and phytohormones are recognized as critical regulators of this coordination, the molecular logic by which their signaling pathways intersect to ensure developmental robustness have remained largely elusive.

**Figure 7:**
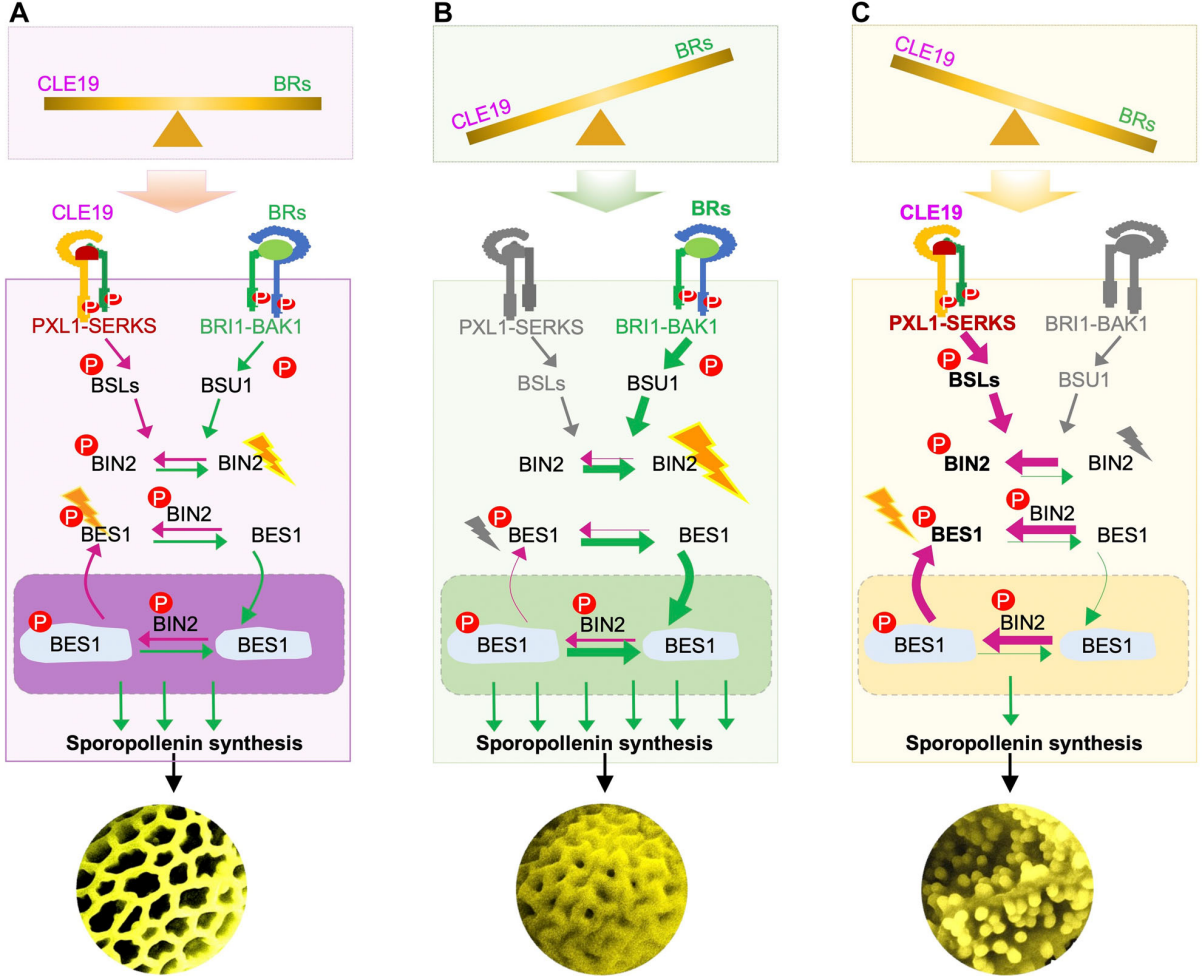
A proposed working model in regulating pollen exine development **(A)** Under normal conditions, balanced CLAVATA3/EMBRYO SURROUNDING REGION‐related 19 (CLE19) and brassinosteroid (BR) signaling coordinate tapetal activity to ensure proper pollen exine formation. CLE19 acts as a spatial “brake” from microspores to restrain BR‐induced transcription via the PXL1–BSLs–BIN2–BES1 phosphorylation cascade. **(B)** In CLE19‐deficient or BR‐hyperactive conditions, unchecked BES1 activation results in excessive or mispatterned exine deposition. This model highlights how local peptide cues and systemic hormones integrate to maintain reproductive robustness. **(C)** Conversely, in CLE19‐overexpression or BR‐deficient backgrounds, excessive BES1 phosphorylation and degradation disrupt transcriptional regulation in tapetal cells, leading to defective pollen exine.

In this study, we define a previously uncharacterized dual‐signaling mechanism in which CLE19 peptide signaling suppresses BR output to maintain pollen exine homeostasis. Building on our previous studies that CLE19 is produced by developing microspores and acts as a diffusible molecular “brake” that restricts excessive transcriptional activation in the tapetum to ensure properly patterned pollen exine formation (Wang et al., 2017; Yu et al., 2023), we now demonstrate that this regulatory role is mediated by direct inhibition of BR signaling through a post‐translational cascade. In contrast to CLE19, BRs act as growth‐promoting steroid hormones that facilitate tapetal development and male fertility by activating TFs such as BES1 (Ye et al., 2010; Chen et al., 2019a, 2019b). Although both CLE19 and BRs are essential, whether and how their opposing activities are mechanistically integrated was not previously understood. Here, we demonstrate that CLE19 suppresses BR output by promoting phosphorylation, nuclear export, and proteasomal degradation of the BR‐responsive transcription factor BES1. This work establishes the CLE19–PXL1–BSL–BIN2–BES1 module as a critical signaling axis that links microspore‐derived peptide cues to intracellular BR signaling dynamics to coordinate tapetal activity and ensure pollen wall integrity (Figure 7).

A major advance of our work lies in uncovering a post‐translational regulatory mechanism by which CLE19 signaling modulates BR output. Through quantitative phosphoproteomic and targeted biochemical analyses, we demonstrated that CLE19 promotes BES1 phosphorylation via activation of BSL phosphatases and BIN2 kinase (1, 2, 3, 4, 5, 6). This post‐translational regulation restricts BES1 nuclear accumulation, thereby attenuating BR‐dependent transcriptional responses. Importantly, CLE19 had no detectable effect on the expression of BR biosynthetic genes, or on the formation of the canonical BRI1–BL–SERK1 receptor complex (Figures 2A, B, S3), supporting the conclusion that CLE19 acts in parallel to or downstream of BRI1. This establishes a distinct regulatory paradigm in which peptide cues modulate hormone output not through biosynthesis or receptor availability but through direct control of transcription factor stability, allowing for rapid and localized developmental reprograming.

We further show that CLE19 promotes BES1 phosphorylation and triggers its nuclear export and degradation (Figure 5), which is in stark contrast to BR signaling that stabilizes BES1 in an unphosphorylated, nuclear‐active state. Notably, we identified two CLE19‐inducible phosphorylation sites, S219 and S223, within the BIN2 phosphorylation domain of BES1. Functional assays reveal that these residues are required for CLE19‐triggered BES1 nuclear export and its functional outcomes in pollen development: phospho‐mimic (S2D) mutants resembled *CLE19‐OX*, while phospho‐dead (S2A) phenocopied *DN‐CLE19* (Figures 6, S6). While the precise structural consequences of these phosphorylation events remain to be further elucidated, our findings demonstrate that specific phosphorylation “codes” can fine‐tune BES1 activity and contribute to spatial and developmental specificity in signal output.

Crucially, our findings suggest that while S219 and S223 are essential for CLE19‐dependent regulation of BES1, they likely are part of a broader phosphorylation landscape orchestrated by BIN2. Given the over 20 known phosphorylation sites on BES1, many of which contribute to its stability and function, it is likely that CLE19 promoted combinational phosphorylation events beyond these two residues, S219 and S223. This multi‐site modification likely fine‐tune BES1's activity in a context‐specific manner, providing an additional layer of regulatory precision in coordinating tapetal development and pollen exine patterning.

To explore whether CLE19‐induced BES1 phosphorylation influences canonical BR signaling responsiveness, we examined the behavior of BES1^S2A^ and BES1^S2D^ variants under exogenous BL treatment. Neither variant showed altered phosphorylation status upon BL exposure, suggesting that S219/S223 are also essential for BR‐mediated BES1 regulation. Although our study focuses primarily on CLE19‐driven modulation, these results imply that CLE19 and BR signaling converge on common regulatory sites within BES1, suggesting a shared downstream integration point of signaling processing. Additionally, the constitutive nuclear localization and functional mimicry of BR overactivation by BES1^S2A^ raise the possibility that this variant might partially bypass defects in upstream components such as *bri1* or *bin2* mutants. Although not directly tested here, this possibility underscores the functional significance of S219/S223 as a node of convergence in CLE19 peptide and BR signaling integration.

In conclusion, we uncover a novel mechanistic framework in which the CLE19 peptide antagonizes BR output through a phosphorylation cascade targeting BES1. This model provides a molecular explanation for how a microspore‐derived peptide signal can locally tune BR signaling to ensure robust and spatially precise control of pollen development. Our findings provide a conceptual foundation for understanding peptide–hormone crosstalk in plants and offer a basis for rational strategies to manipulate developmental signaling for improved fertility, resilience, and developmental precision in crop breeding.

## MATERIALS AND METHODS

### Plant growth conditions and materials

The *Arabidopsis thaliana* Columbia (Col‐0), Lansberg *erecta* (*Ler*), and C24 ecotypes were utilized in this study. Previously described mutants include *bri1‐301* (Xu et al., 2008), *bin2‐1* (Li et al., 2001), *bin2‐3 bil1 bil2* (Yan et al., 2009), *BES1‐RNAi* (Yin et al., 2005), *bes1‐D* (Yin et al., 2005), and *bsl1 bsl2 bsl3* (Guo et al., 2022). After 2 d of vernalization at 4°C, all WT and mutant seeds were planted and grown in soil at 22°C under a 16 h‐light/8 h‐dark photoperiod. *Nicotiana benthamiana* plants were cultivated in a greenhouse under previously reported conditions.

### Parallel phosphoproteomic analysis

**Protein extraction and digestion:** Stage 1–10 flowers from Col‐0, *DN‐CLE19*, C24, *CLE19‐OX* plants were collected, and immediately frozen in lipid nitrogen. Two biological replicates of each genotype were gathered. Then proteins were extracted from each sample and subjected to trypsin digestion as previously reported (Ye et al., 2015).

**iTRAQ labeling:** Approximately 100 μg of peptides from each sample were labeled separately using the iTRAQ (Applied Biosystems, Foster City, CA, USA) standard protocol for the 4‐plex kit. For the *DN‐CLE19* case, the two replicates of *DN‐CLE19* protein samples were labeled with 114 and 116, and the two Col‐0 replicates were labeled with 115 and 117, respectively. For the *CLE19‐OX* case, the two replicates of *CLE19‐OX* protein samples were labeled with 114 and 116, and the two C24 replicates were labeled with 115 and 117, respectively.

**Phosphopeptides enrichment:** Peptide mixture from 80 μg of protein (approximately 1/5 of the total protein) were used for phosphoproteomic analysis. Phosphopeptides were enriched using a TiO_2_ micro‐column following previously described protocols. The digests were vacuum dried and resuspended with TiO_2_ loading buffer (1 mol/L glycolic acid in 80% acetonitrile (can), 1% trifluoracetic acid (TFA)) and applied onto the TiO_2_ micro‐column. After washing four times with 20 μL loading buffer and at least three times with 20 μL washing buffer (80% ACN, 1% TFA), the bound peptides were eluted twice with 20 μL elution buffer 1 (2 mol/L NH_3_•H_2_O), and with 2 μL elution buffer 2 (1 mol/L NH_3_•H_2_O in 40% ACN).

**Nano ultra‐high performance liquid chromatology – tandem MS (UHPLC‐MS/MS) analysis:** The peptides mixtures were desalted before LC‐MS/MS analysis, which was performed using an LTQ‐Orbitrap Elite (ThermoFisher) coupled with an Easy nLC 1,000. The LC solvents are 0.1% Formic Acid (FA) in H_2_O (Solvent A) and 0.1% FA in 95% ACN (Solvent B). The peptide separation was accomplished by three‐step elution: 2%–35% Solvent B for 200 min, 35%–90% Solvent B for 10 min, 90% Solvent B for 5 min, 2% Solvent B for 2 min, and 2% for 13 min. The peptide ions were detected in the Orbitrap mass spectrometer and up to 15 of the most intense peptide ions (> 5,000 counts) were selected and fragmented by MS/MS using multistage activation (MSA) in the linear ion trap (Palumbo and Reid, 2008).

**Database search and data analysis**: Raw data were processed using Proteome Discoverer software (Version 1.4; ThermoFisher, Germany) and searched against the Arabidopsis database (FASTA data downloaded from ftp://ftp.arabidopsis.org/home/tair/Sequences/blast_datasets/TAIR10_blastsets/TAIR10_pep_20110103_representative_gene_model_updated) using an in‐house Mascot server (Version 2.3.02; Matrix Science, London, UK).

The following parameters were specified in the protein database searches: carbamidomethyl‐cysteine, iTRAQ 4‐plex (N‐term), iTRAQ 4‐plex (K) as a fixed modification; and protein *N*‐acetylation, oxidized methionine, iTRAQ 4‐plex (Y) permitted as variable modifications (in phosphoproteomics, phospho_STY (serine, threonine, and tyrosine)) were added as mentioned in the article. Peptide identification was achieved by using the following requirements: the expectation value (*P*) was lower than 0.05 and the peptides were ranked as No. 1 by the Mascot search engine (Matrix Science). Identified peptides were further validated with Target Decoy PSM validator and phosphorylation sites were evaluated with phosphoRS3.0.

Only unique peptides in which confidence was more than 95% were contained in iTRAQ labeling quantification. For the two biological replicates, the average level was calculated. The fold change was the ratio of protein abundance between mutants and control. Proteins with a fold change (mutants: WT, *m*/*W*) larger than 1.2 (Log_2_ (*m*/*W*) ≥ Log_2_ (1.2) or Log_2_ (*m*/*W*) ≤ −Log_2_ (1.2)) in some of the three technical replicates A, B, and C were considered to be significantly differentially expressed.

### Generation of constructs and transgenic plants

The full‐length coding sequence of BES1 was amplified from complementary DNA (cDNA) of Col‐0 plants and subjected to site‐directed mutagenesis to generate the S219AS223A and S219DS223D mutations using a mutagenesis kit (FM111, Transgen, CN, USA). All Primers used for vector construction are listed in Table S1. Subsequently, BES1, BES1^S2A^, and BES1^S2D^ were cloned into the pGWB441 vector using the Gateway cloning system (the BP Clonase II Enzyme (11789100; Invitrogen, USA) and the LR Clonase II Enzyme (11791100; Invitrogen, USA)), resulting in the 35S:BES1‐EYFP, 35S:BES1^S2A^‐EYFP and 35S:BES1^S2D^‐EYFP constructs. These constructs were introduced into Col‐0 and *CLE19‐OX* plants via *Agrobacterium tumefaciens*‐mediated floral transformation. T3 transgenic plants were selected and used for subsequent analyses.

### Phenotypic analysis of flowers and anthers

Plants were photographed with a digital camera (Canon, Japan), and images of flowers were captured. Stage 12 anthers were stained and observed as previously reported. Inflorescences from WT and mutant plants were fixed in formaldehyde–acetic acid–ethanol (FAA) solution, embedded in Technovit 7100 resin (HeraeusKulzer GmbH, Germany) solution, and sectioned semi‐thin as described (Chang et al., 2014). The sections were then stained with 0.05% Toluidine Blue O for 5 min and photographed (Chang et al., 2014).

### RNA purification and qRT‐PCR analysis

Arabidopsis inflorescences of WT and mutant plants were collected and quickly frozen in liquid nitrogen. Total RNA was extracted using Invitrogen TRIZOL® reagent and digested by DNAse I (Roche Diagnostics, Shanghai, China). One microgram of RNA was reverse‐transcripted for first‐strand cDNA synthesis following genomic DNA (gDNA) eraser (Takara, Shanghai, China) digestion. Real‐time PCR experiments were carried out on the ABI Step One Plus™ Real‐Time system using TAKARA SYB^R^® premix *Ex Taq™ II* (TAKARA, Dalian, China). Expression levels were calculated using the ΔΔ threshold cycle (*C*_*t*_) method with Actin (AT3G18780) as the internal control. The qRT‐PCR primers are listed in Table S1.

### Luciferase complementation imaging assay

The full‐length coding region of PXL1 was cloned into the pCCL vector, fusing it with C‐terminal Cluc. Similarly, the full‐length coding sequences of BSL1/2/3 and BSU1 were cloned into the pHNL vector with C‐terminal NLuc fusion. All constructs were introduced into *Agrobacterium tumefaciens* strain GV3101. Monoclonal colonies were cultured in liquid Luria‐Bertani (LB) medium. The concentration of the bacteria was measured by spectrophotometer. Agrobacterium cultures carrying different combination constructs were resuspended with injection buffer (150 μmol/L acetosyringone, 10 mmol/L MgCl_2_ and 10 mmol/L 2‐(N‐morpholino)ethanesulfonic acid (MES), pH 5.6) and mixed at 1:1 volume ratio. The mixtures were infiltrated into 4‐week‐old *Nicotiana benthamiana* leaves. After 48 h, leaves were sprayed with 0.25 μmol/L luciferin substrate and kept in the dark for 5–10 min before luminescence was captured with an LB985 NightShade imaging system (Berthhold Tech).

### *In vivo* phosphorylation assay

The full‐length coding region of BSL1 was inserted into the pCAMBIA1306–FLAG vector to obtain the pCAMBIA1306–BSL1–FLAG construct, and full‐length PXL1 coding region was inserted into the pCAMBIA1306–MYC vector to obtain the pCAMBIA1306–PXL1–MYC construct. pCAMBIA1306–BSL1–FLAG and pCAMBIA1306–PXL1–MYC were transformed into *Agrobacterium tumefaciens* strain GV3101. Monoclonal bacteria were then picked and cultured in liquid medium; each Agrobacterium was resuspended with injection buffer (150 μmol/L acetosyringone, 10 mmol/L MgCl_2_ and 10 mmol/L MES, pH 5.6) and mixed at 1:1 volume ratio, and different combinations of Agrobacterium were injected into 4‐week‐old tobacco leaves. After 2 d culture, 10 μmol/L of CLE19 peptide (A peptide synthesize) was injected into tobacco leaves. The total protein of the leaves was extracted using protein extract buffer (50 mmol/L Tris‐HCl, pH 7.5, 150 mmol/L NaCl, 5 mmol/L ethylenediaminetetraacetic acid, 1% SDS) supplemented with protease and phosphatase inhibitors. Protein extracts were incubated with anti‐FLAG beads at 4°C for 3 h, and a combination of CLE19 injections was sampled after 1 h. Proteins were resolved via SDS‐PAGE and analyzed by western blotting using anti‐MYC (GNI) and anti‐FLAG (GNI) antibody. Anti‐pS/T (Abmart) antibody was used to assess BSL1 phosphorylation status in the presence or absence of CLE19 peptide.

### Accession numbers

The original RNA‐seq, quantitative proteomic and phosphoproteomic data from this article have been submitted to the National Center for Biotechnology Information Gene Expression Omnibus database under accession number GS394607. Sequence data from this article can be found in the Arabidopsis Genome Initiative database under the following accession numbers: *CLE19* (AT3G24225), *DYT1* (AT4G21330), *AMS* (AT2G16910), *MYB35/TDF1* (AT3G28470), *MYB80/MYB103* (AT5G56110), *MS1* (AT5G22260), *BES1* (AT1G19350), *BRI1* (AT4G39400), *SERK1* (AT1G71830), *BIN2* (AT4G18710), *BSL1*(AT4G03080), *BSL2*(AT1G08420), *BSL3*(AT2G27210), *DWF1* (AT3G19820), *DWF4* (AT3G50660), *DWF5* (AT1G50430), *DWF7* (AT3G02580), *CPD* (AT5G05690), *DET2* (AT2G38050), *ROT3* (AT4G36380), *CYP90D1* (AT3G13730), and *ACT2* (AT3G18780).

### Quantification and statistical analysis

All the statistical analyses in this study were performed by GraphPad Prism 10. All the statistical details of experiments, including the statistical tests used, the exact values of sample numbers, and precision measures, can be found in the figure legends. All comparisons and *P*‐values are indicated in the figures.

## AUTHOR CONTRIBUTIONS

F.C. and H.M conceived this project; F.C., S.W., S.Z., and Y.Y. designed the research. S.Z., S.W., and Y.Y. performed most of the experiments and data organization, with assistance from J.W., H.L., Y.L., and Y. Z.; M.L., J.L. and J.Y. conducted the proteomic analyses. M.L. and J.W. also carried out the transcriptomic analyses. W.S. carried out the gel filtration analysis. F.C., S.W., S.Z., and Y.Y. analyzed the data and wrote the manuscript. All authors read and approved of the manuscript.

## Supporting information

Additional Supporting Information may be found online in the supporting information tab for this article: http://onlinelibrary.wiley.com/doi/10.1111/jipb.70024/suppinfo

**Dataset S1.** The 1,091 proteins with significantly different phosphorylation levels in *DN‐CLE19*

**Dataset S2.** The 1,071 proteins with significantly different phosphorylation levels in *CLE19‐OX*

**Dataset S3.** The 428 genes presented in both the CORE‐CLE and CORE‐BR gene sets

**Figure S1.** The definition of the CORE‐CLE and CORE‐BR genes
**Figure S2.** The top 12 significantly enriched Gene Ontology terms in the 371 overlapping genes
**Figure S3.** CLE19 does not affect BR biosynthesis
**Figure S4.** RNA *in situ* hybridization analyses showed the expression patterns of *BRL1*, *BSL2*, *BSL3*, *BIN2*, and *BES1* in WT anthers
**Figure S5.** The expression analysis of the transcripts of BES1‐S and BES1‐L in Stage 4–10 anthers
**Figure S6.** S‐to‐A mutation blocked CLE19‐induced BES1 nuclear‐to‐cytosol export

**Table S1.** Primers used in this study

## References

[jipb70024-bib-0001] Chang, F. , Wang, Y. , Wang, S. , and Ma, H. (2011). Molecular control of microsporogenesis in *Arabidopsis* . Curr. Opin. Plant Biol. 14: 66–73. 21145279 10.1016/j.pbi.2010.11.001

[jipb70024-bib-0002] Chang, F. , Zhang, Z. , Jin, Y. , and Ma, H. (2014). Cell biological analyses of anther morphogenesis and pollen viability in *Arabidopsis* and rice. Methods Mol. Biol. 1110: 203–216. 24395258 10.1007/978-1-4614-9408-9_9

[jipb70024-bib-0003] Chen, L.G. , Gao, Z. , Zhao, Z. , Liu, X. , Li, Y. , Zhang, Y. , Liu, X. , Sun, Y. , and Tang, W. (2019a). BZR1 family transcription factors function redundantly and indispensably in BR signaling but exhibit BRI1‐independent function in regulating anther development in *Arabidopsis* . Mol. Plant 12: 1408–1415. 31229643 10.1016/j.molp.2019.06.006

[jipb70024-bib-0004] Chen, W. , Lv, M. , Wang, Y. , Wang, P.‐A. , Cui, Y. , Li, M. , Wang, R. , Gou, X. , and Li, J. (2019b). BES1 is activated by EMS1‐TPD1‐SERK1/2‐mediated signaling to control tapetum development in *Arabidopsis thaliana* . Nat. Commun. 10: 4164. 31519953 10.1038/s41467-019-12118-4 PMC6744560

[jipb70024-bib-0005] Clouse, S.D. , Langford, M. , and McMorris, T.C. (1996). A brassinosteroid‐insensitive mutant in *Arabidopsis thaliana* exhibits multiple defects in growth and development. Plant Physiol. 111: 671–678. 8754677 10.1104/pp.111.3.671 PMC157882

[jipb70024-bib-0006] Cui, J. , You, C. , Zhu, E. , Huang, Q. , Ma, H. , and Chang, F. (2016). Feedback regulation of DYT1 by interactions with downstream bHLH factors promotes DYT1 nuclear localization and anther development. Plant Cell 28: 1078–1093. 27113773 10.1105/tpc.15.00986 PMC4904671

[jipb70024-bib-0007] Feng, B. , Lu, D. , Ma, X. , Peng, Y. , Sun, Y. , Ning, G. , and Ma, H. (2012). Regulation of the *Arabidopsis* anther transcriptome by DYT1 for pollen development. Plant J. 72: 612–624. 22775442 10.1111/j.1365-313X.2012.05104.x

[jipb70024-bib-0009] Ge, X. , Chang, F. , and Ma, H. (2010). Signaling and transcriptional control of reproductive development in *Arabidopsis* . Curr. Biol. 20: R988–R997. 21093795 10.1016/j.cub.2010.09.040

[jipb70024-bib-0010] Gómez, J.F. , Talle, B. , and Wilson, Z.A. (2015). Anther and pollen development: A conserved developmental pathway. J. Integr. Plant Biol. 57: 876–891. 26310290 10.1111/jipb.12425 PMC4794635

[jipb70024-bib-0011] Guo, X. , Ding, X. , and Dong, J. (2022). Dichotomy of the BSL phosphatase signaling spatially regulates MAPK components in stomatal fate determination. Nat. Commun. 13: 2438. 35508457 10.1038/s41467-022-30254-2 PMC9068801

[jipb70024-bib-0012] Heslop‐Harrison, J. (1971). Wall pattern formation in angiosperm microsporogenesis. Symp. Soc. Exp. Biol. 25: 277–300. 4940549

[jipb70024-bib-0013] Hsieh, K. , and Huang, A.H.C. (2007). Tapetosomes in *Brassica tapetum* accumulate endoplasmic reticulum–derived flavonoids and alkanes for delivery to the pollen surface. Plant Cell 19: 582–596. 17307923 10.1105/tpc.106.049049 PMC1867322

[jipb70024-bib-0014] Jiang, J. , Zhang, C. , and Wang, X. (2015). A recently evolved isoform of the transcription factor BES1 promotes brassinosteroid signaling and development in *Arabidopsis thaliana* . Plant Cell 27: 361–374. 25649439 10.1105/tpc.114.133678 PMC4456931

[jipb70024-bib-0015] Lai, Z. , Wang, J. , Fu, Y. , Wang, M. , Ma, H. , Peng, S. , and Chang, F. (2023). Revealing the role of CCoAOMT1: Fine‐tuning bHLH transcription factors for optimal anther development. Sci. China: Life Sci. 67: 565–578. 38097889 10.1007/s11427-023-2461-0

[jipb70024-bib-0016] Lai, Z. , Wang, J. , Peng, S.‐Q. , and Chang, F. (2022). bHLH010/089 transcription factors control pollen wall development via specific transcriptional and metabolic networks in *Arabidopsis thaliana* . Int. J. Mol. Sci. 23: 11683. 36232985 10.3390/ijms231911683 PMC9570398

[jipb70024-bib-0017] Li, J. , Nam, K.H. , Vafeados, D. , and Chory, J. (2001). BIN2, a new brassinosteroid‐insensitive locus in *Arabidopsis* . Plant Physiol. 127: 14–22. 11553730 10.1104/pp.127.1.14 PMC117958

[jipb70024-bib-0018] Li, X. , Li, J. , Zabed, H.M. , Li, J. , Xiong, M. , Shi, H. , and Li, J. (2025). *Manipulating brassinosteroid signaling pathway to genetically improve horticultural plants*. aBIOTECH. 10.1007/s42994-025-00201-y PMC12238443 40641636

[jipb70024-bib-0019] Mariani, C. , Beuckeleer, M.D. , Truettner, J. , Leemans, J. , and Goldberg, R.B. (1990). Induction of male sterility in plants by a chimaeric ribonuclease gene. Nature 347: 737–741.

[jipb70024-bib-0020] Nolan, T.M. , Vukašinović, N. , Liu, D. , Russinova, E. , and Yin, Y. (2020). Brassinosteroids: Multidimensional regulators of plant growth, development, and stress responses. Plant Cell 32: 295–318. 31776234 10.1105/tpc.19.00335 PMC7008487

[jipb70024-bib-0022] Palumbo, A.M. , and Reid, G.E. (2008). Evaluation of gas‐phase rearrangement and competing fragmentation reactions on protein phosphorylation site assignment using collision induced dissociation‐MS/MS and MS3. Anal. Chem. 80: 9735–9747. 19012417 10.1021/ac801768s

[jipb70024-bib-0024] Piffanelli, P. , Ross, J.H.E. , and Murphy, D.J. (1998). Biogenesis and function of the lipidic structures of pollen grains. Plant Reprod. 11: 65–80.

[jipb70024-bib-0025] Stieglitz, H. , and Stern, H. (1973). Regulation of beta‐1,3‐glucanase activity in developing anthers of *Lilium* . Dev. Biol. 34: 169–173. 4787601 10.1016/0012-1606(73)90347-3

[jipb70024-bib-0026] Wang, S. , Lu, J. , Song, X.F. , Ren, S.C. , You, C. , Xu, J. , Liu, C.M. , Ma, H. , and Chang, F. (2017). Cytological and transcriptomic analyses reveal important roles of CLE19 in pollen exine formation. Plant Physiol. 175: 1186–1202. 28916592 10.1104/pp.17.00439 PMC5664459

[jipb70024-bib-0027] Xu, J. , Ding, Z. , Vizcay‐Barrena, G. , Shi, J. , Liang, W. , Yuan, Z. , Werck‐Reichhart, D. , Schreiber, L. , Wilson, Z.A. , and Zhang, D. (2014). ABORTED MICROSPORES acts as a master regulator of pollen wall formation in *Arabidopsis* . Plant Cell 26: 1544–1556. 24781116 10.1105/tpc.114.122986 PMC4036570

[jipb70024-bib-0028] Xu, J. , Yang, C. , Yuan, Z. , Zhang, D. , Gondwe, M.Y. , Ding, Z. , Liang, W. , Zhang, D. , and Wilson, Z.A. (2010). The ABORTED MICROSPORES regulatory network is required for postmeiotic male reproductive development in *Arabidopsis thaliana* . Plant Cell 22: 91–107. 20118226 10.1105/tpc.109.071803 PMC2828693

[jipb70024-bib-0029] Xu, W. , Huang, J. , Li, B. , Li, J. , and Wang, Y. (2008). Is kinase activity essential for biological functions of BRI1? Cell Res. 18: 472–478. 18332904 10.1038/cr.2008.36

[jipb70024-bib-0030] Yan, Z. , Zhao, J. , Peng, P. , Chihara, R.K. , and Li, J. (2009). BIN2 functions redundantly with other Arabidopsis GSK3‐Like kinases to regulate brassinosteroid signaling. Plant Physiol. 150: 710–721. 19395409 10.1104/pp.109.138099 PMC2689954

[jipb70024-bib-0031] Ye, J. , Zhang, Z. , Long, H. , Zhang, Z. , Hong, Y. , Zhang, X. , You, C. , Liang, W. , Ma, H. , and Lu, P. (2015). Proteomic and phosphoproteomic analyses reveal extensive phosphorylation of regulatory proteins in developing rice anthers. Plant J. 84: 527–544. 26360816 10.1111/tpj.13019

[jipb70024-bib-0032] Ye, Q. , Zhu, W. , Li, L. , Zhang, S. , Yin, Y. , Ma, H. , and Wang, X. (2010). Brassinosteroids control male fertility by regulating the expression of key genes involved in *Arabidopsis* anther and pollen development. Proc. Natl. Acad. Sci. U.S.A. 107: 6100–6105. 20231470 10.1073/pnas.0912333107 PMC2851861

[jipb70024-bib-0033] Yin, Y. , Vafeados, D. , Tao, Y. , Yoshida, S. , Asami, T. , and Chory, J. (2005). A new class of transcription factors mediates brassinosteroid‐regulated gene expression in *Arabidopsis* . Cell 120: 249–259. 15680330 10.1016/j.cell.2004.11.044

[jipb70024-bib-0034] Yin, Y. , Wang, Z.‐Y. , Mora‐Garcia, S. , Li, J. , Yoshida, S. , Asami, T. , and Chory, J. (2002). BES1 accumulates in the nucleus in response to brassinosteroids to regulate gene expression and promote stem elongation. Cell 109: 181–191. 12007405 10.1016/s0092-8674(02)00721-3

[jipb70024-bib-0035] Yu, Y. , Song, W. , Zhai, N. , Zhang, S. , Wang, J. , Wang, S. , Liu, W. , Huang, C.‐H. , Ma, H. , Chai, J. , et al. (2023). PXL1 and SERKs act as receptor–coreceptor complexes for the CLE19 peptide to regulate pollen development. Nat. Commun. 14: 3307. 37286549 10.1038/s41467-023-39074-4 PMC10247778

[jipb70024-bib-0036] Zhang, W. , Sun, Y. , Timofejeva, L. , Chen, C. , Grossniklaus, U. , and Ma, H. (2006). Regulation of *Arabidopsis* tapetum development and function by DYSFUNCTIONAL TAPETUM1 (DYT1) encoding a putative bHLH transcription factor. Development 133: 3085–3095. 16831835 10.1242/dev.02463

[jipb70024-bib-0037] Zhu, E. , You, C. , Wang, S. , Cui, J. , Niu, B. , Wang, Y. , Qi, J. , Ma, H. , and Chang, F. (2015). The DYT1‐interacting proteins bHLH010, bHLH089 and bHLH091 are redundantly required for *Arabidopsis* anther development and transcriptome. Plant J. 83: 976–990. 26216374 10.1111/tpj.12942

[jipb70024-bib-0038] Zhu, J. , Chen, H. , Li, H. , Gao, J.F. , Jiang, H. , Wang, C. , Guan, Y.F. , and Yang, Z.N. (2008). Defective in Tapetal Development and Function 1 is essential for anther development and tapetal function for microspore maturation in *Arabidopsis* . Plant J. 55: 266–277. 18397379 10.1111/j.1365-313X.2008.03500.x

[jipb70024-bib-0039] Zhu, J. , Lou, Y. , Xu, X. , and Yang, Z.‐N. (2011). A genetic pathway for tapetum development and function in *Arabidopsis* . J. Integr. Plant Biol. 53: 892–900. 21957980 10.1111/j.1744-7909.2011.01078.x

