# Supplementary material for: CLE19 suppresses brassinosteroid signaling output via the BSL‐BIN2 module to maintain BES1 activity and pollen exine patterning in Arabidopsis
Source: J Integr Plant Biol. 2025 Aug 28;67(12):3216–30. doi: 10.1111/jipb.70024 (PMC12678687; doi:10.1111/jipb.70024)
Supplement: Supplementary file 4 — Figure S1. The definition of the CORE‐CLE and CORE‐BR genes Figure S2. The top 12 significantly enriched Gene Ontology terms in the 371 overlapping genes Figure S3. CLE19 does not affect BR biosynthesis Figure S4. RNA in situ hybridization analyses showed the expression patterns of BRL1, BSL2, BSL3, BIN2, and BES1 in WT anthers Figure S5. The expression analysis of the transcripts of BES1‐S and BES1‐L in Stage 4–10 anthers Figure S6. S‐to‐A mutation blocked CLE19‐induced BES1 nuclear‐to‐cytosol export [file JIPB-67-3216-s003.docx]

**Supplemental Figures S1–S6:**

**Figure S1. The definition of the CORE-CLE and CORE-BR genes**

**Figure S2. The top 12 significantly enriched GO terms in the 371 overlapping genes**

**Figure S3. CLE19 does not affect BR biosynthesis**

**Figure S4. RNA *in situ* hybridization analyses showed the expression patterns of *BRL1*, *BSL2*, *BSL3*, *BIN2*, and *BES1* in WT anthers**

**Figure S5. The expression analysis of the transcripts of BES1-S and BES1-L in stage 4-10 anthers**

**Figure S6. S-to-A mutation blocked CLE19-induced BES1 nuclear-to-cytosol export.**

**
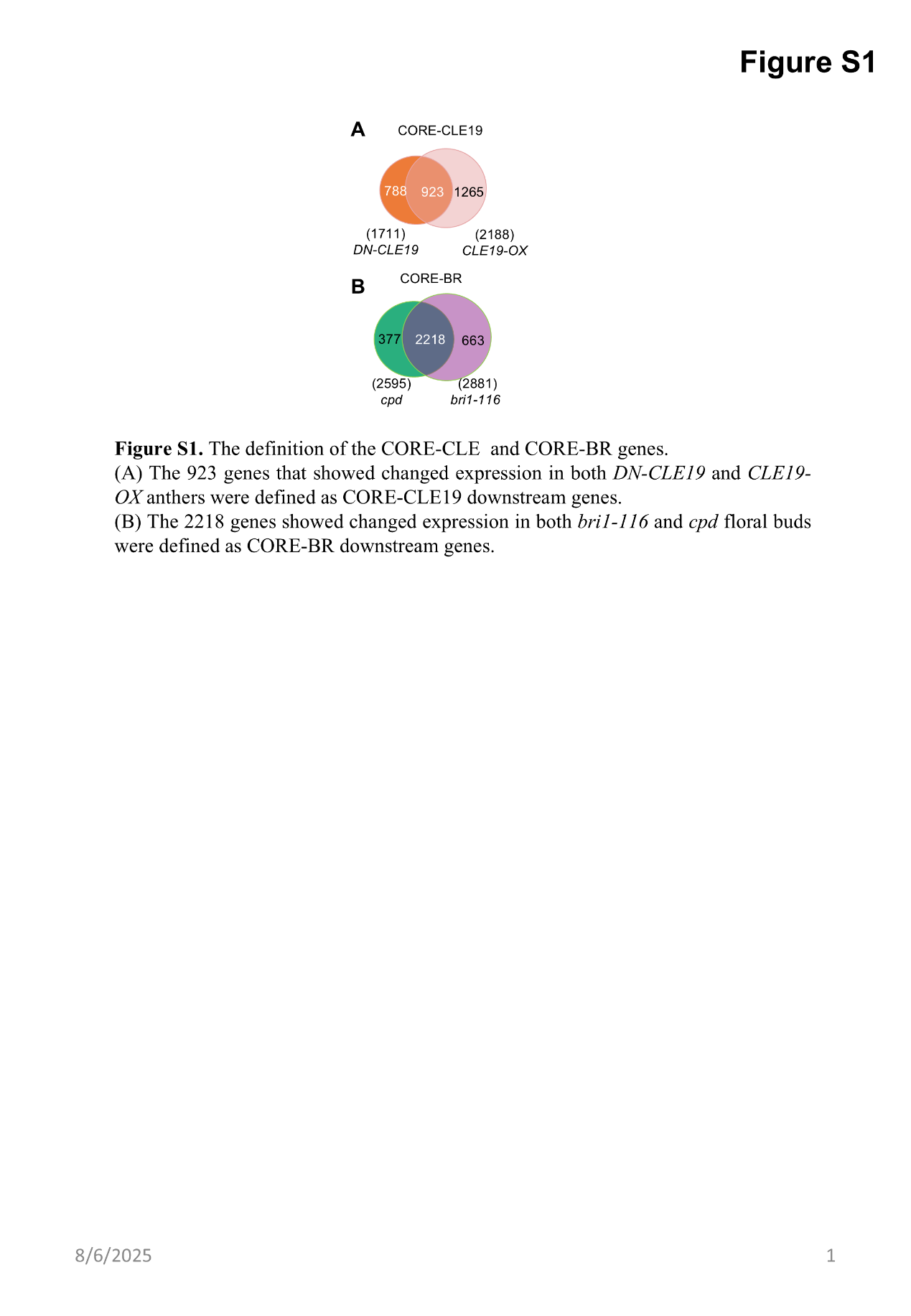
**

**Figure S1. The definition of the CORE-CLE and CORE-BR genes**

(**A**) The 923 genes that showed changed expression in both *DN-CLE19* and *CLE19-OX* anthers were defined as CORE-CLE19 downstream genes.

(**B**) The 2218 genes showed changed expression in both *bri1-116* and *cpd* floral buds were defined as CORE-BR downstream genes.


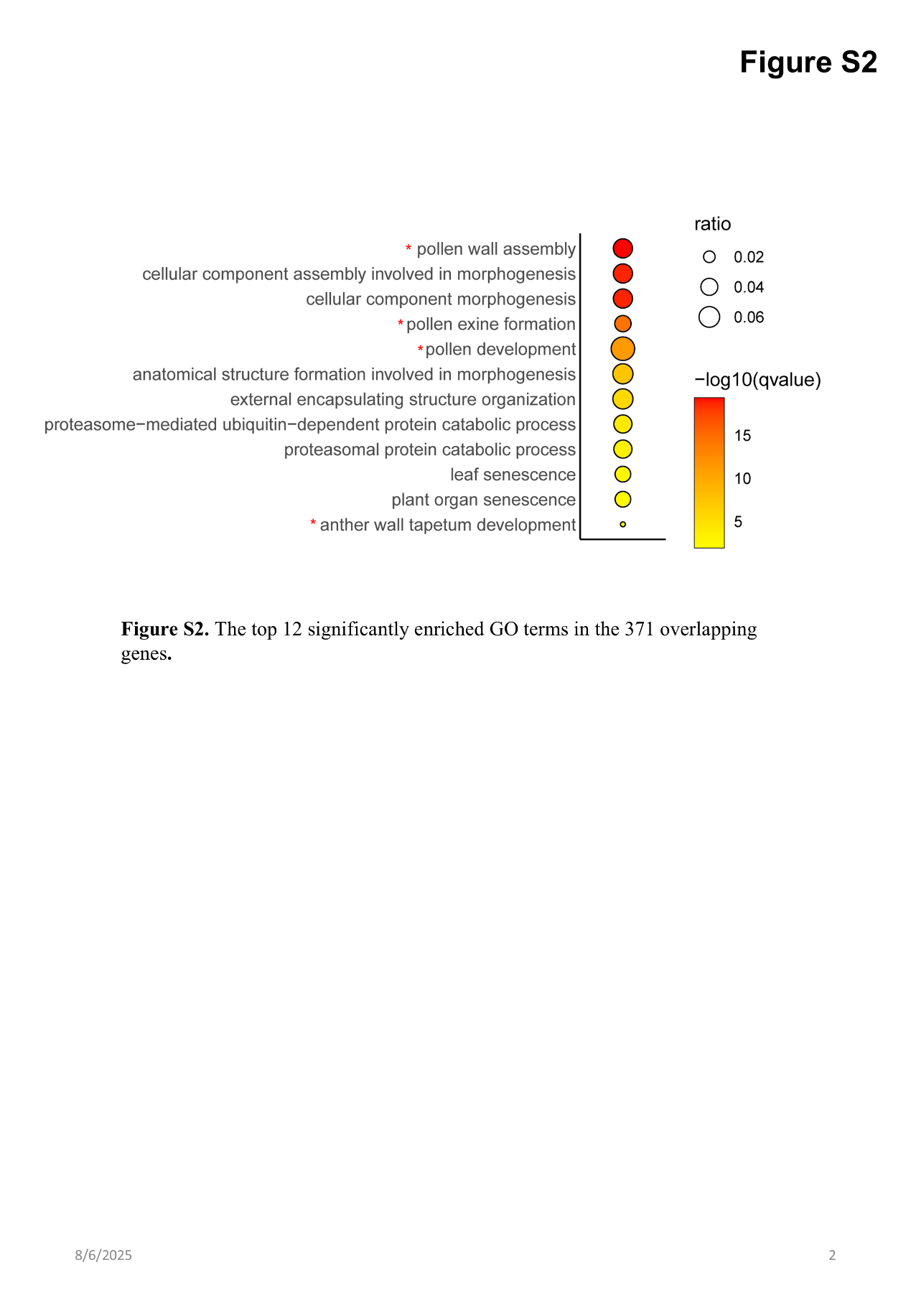


**Figure S2. The top 12 significantly enriched GO terms in the 371 overlapping genes**


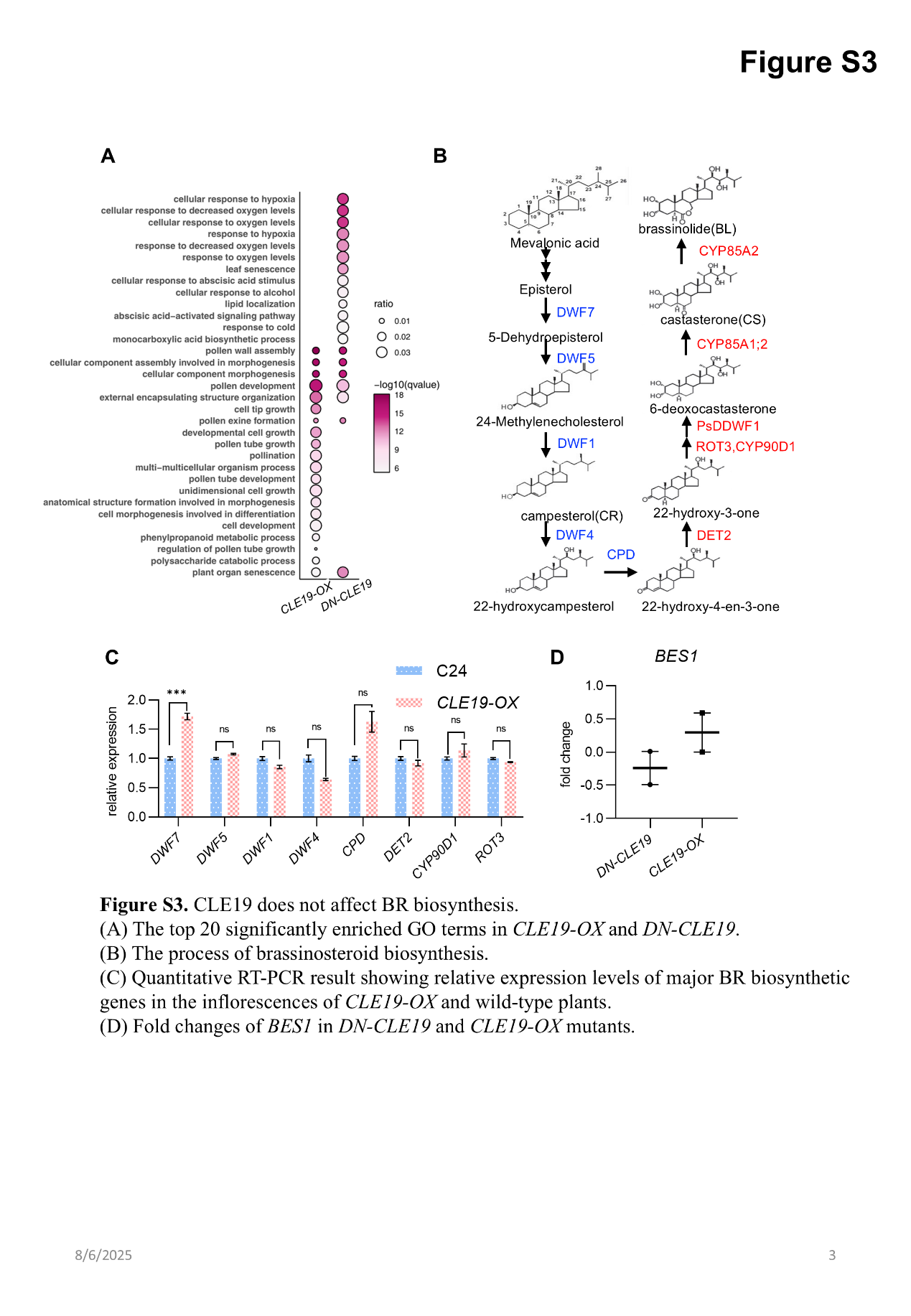


**Figure S3. CLE19 does not affect BR biosynthesis**

(**A**) The top 20 significantly enriched GO terms in *CLE19-OX* and *DN-CLE19*.

(**B**) The process of brassinosteroid biosynthesis.

(**C**) Quantitative RT-PCR result showing relative expression levels of major BR biosynthetic genes in the inflorescences of CLE19-OX and wild-type plants.

(**D**) Fold changes of *BES1* in *DN-CLE19* and *CLE19-OX* mutants.


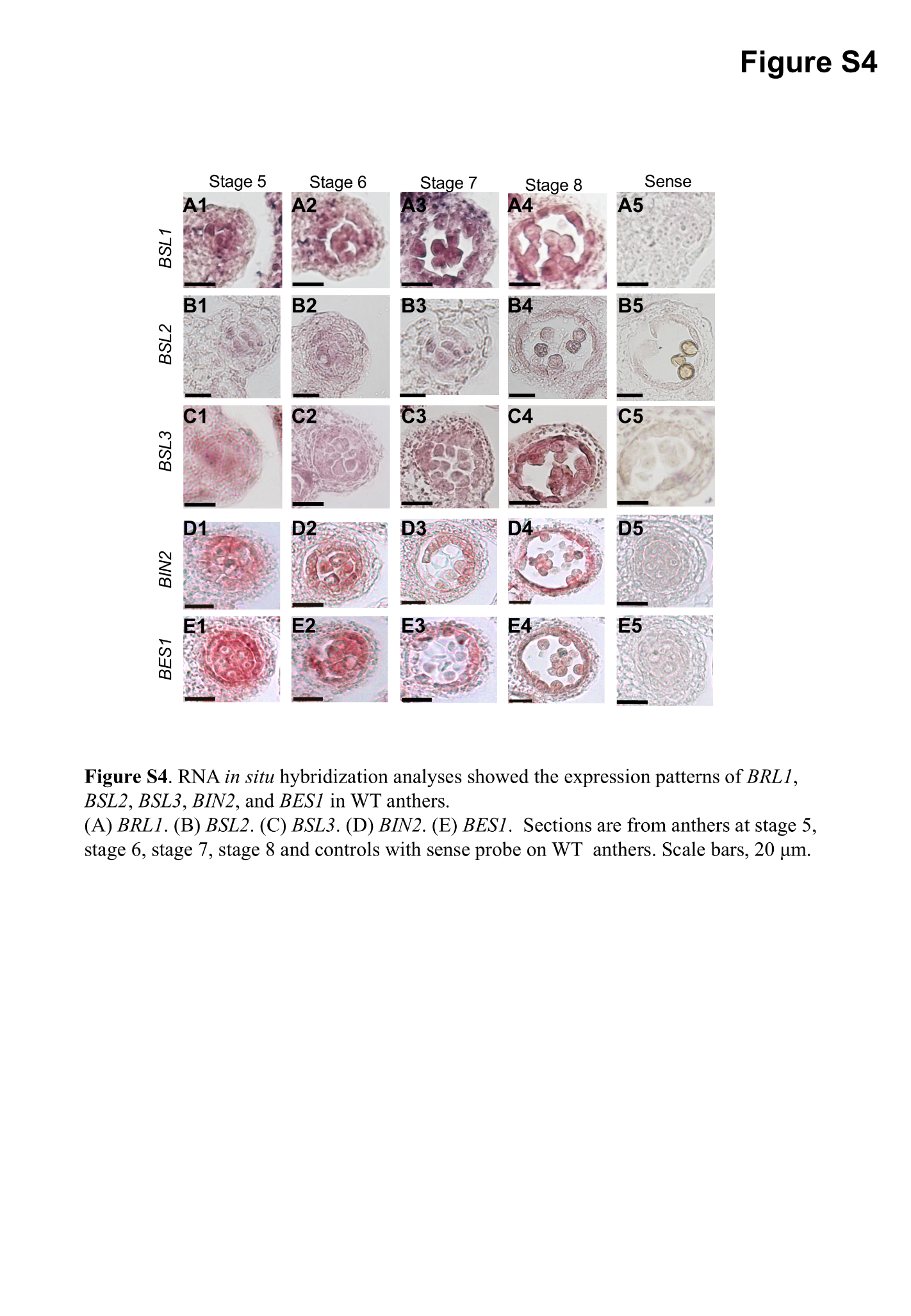


**Figure S4. RNA *in situ* hybridization analyses showed the expression patterns of *BRL1*, *BSL2*, *BSL3*, *BIN2*, and *BES1* in WT anthers**

(**A**) *BRL1*. (**B**) *BSL2*. (**C**) *BSL3*. (**D**) *BIN2*. (**E**) *BES1*. Sections are from anthers at stage 5, stage 6, stage 7, stage 8 and controls with sense probe on WT anthers. Scale bars, 20 μm.


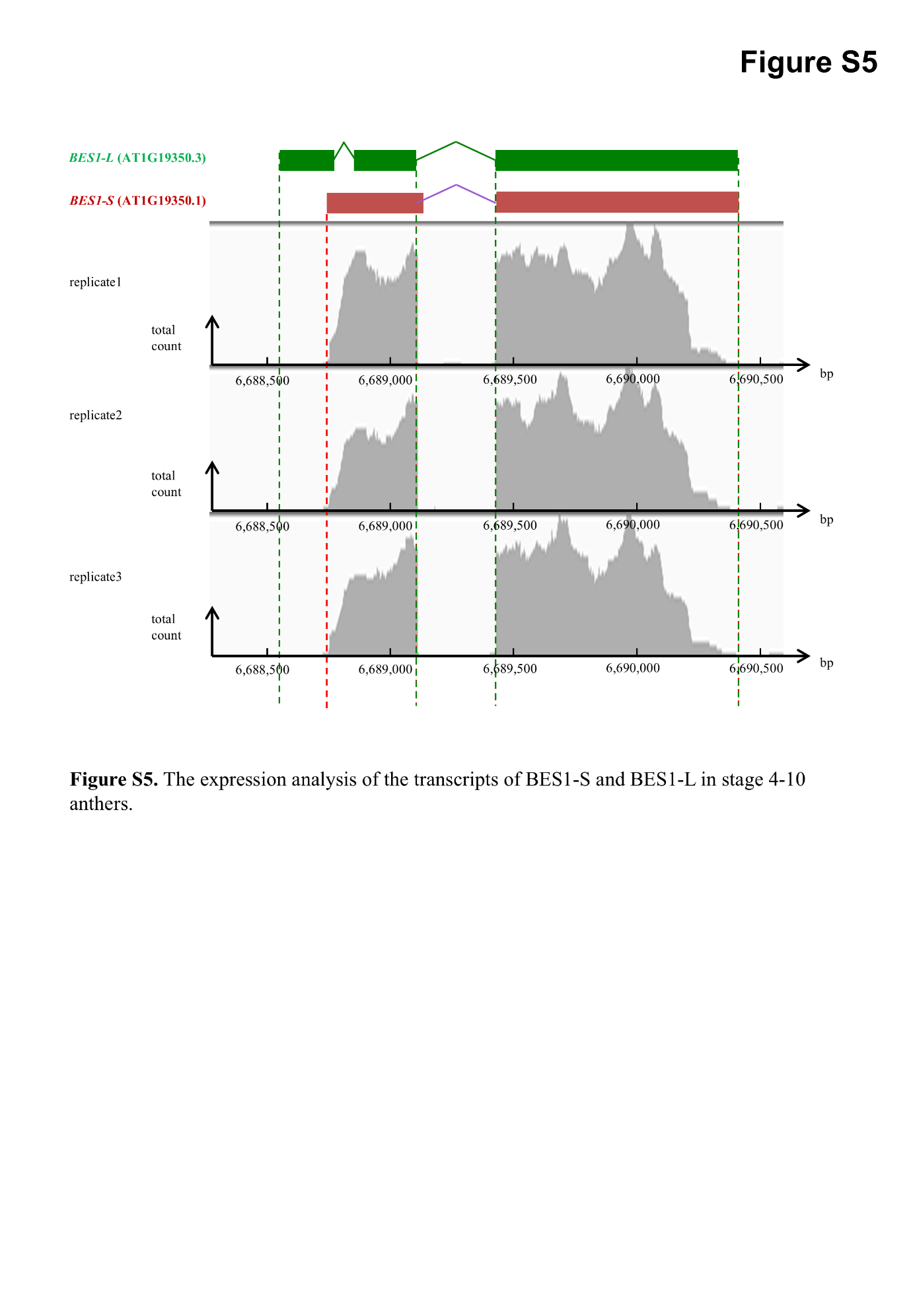


**Figure S5. The expression analysis of the transcripts of BES1-S and BES1-L in stage 4-10 anthers**


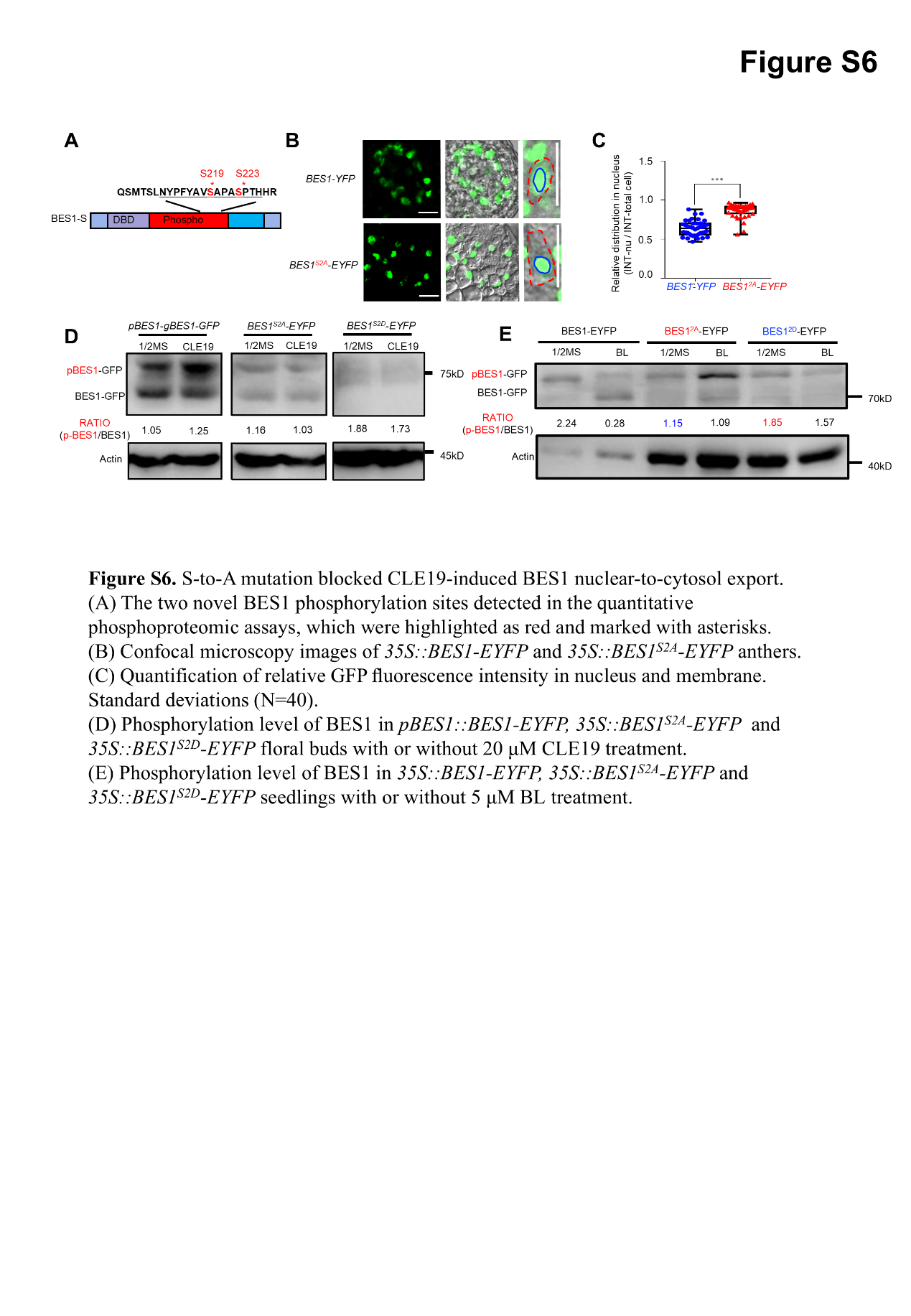


**Figure S6. S-to-A mutation blocked CLE19-induced BES1 nuclear-to-cytosol export**

(**A**) The two novel BES1 phosphorylation sites detected in the quantitative phosphoproteomic assays, which were highlighted as red and marked with asterisks.

(**B**) Confocal microscopy images of *35S::BES1-EYFP* and *35S::BES1^S2A^-EYFP* anthers.

(**C**) The ratio of nuclear fluorescence intensity to total cellular fluorescence for individual cells (nuclear/total-cell) was quantified in individual tapetal cells **to evaluate the impact of the S2A mutation on BES1 subcellular localization while minimizing cell-to-cell variation**. Standard deviations (N=40). (**D**) Phosphorylation level of BES1 in *pBES1::BES1-EYFP,* *35S::BES1^S2A^-EYFP* and *35S::BES1^S2D^-EYFP* floral buds with or without 20 μM CLE19 treatment.

(**E**) Phosphorylation level of BES1 in *35S::BES1-EYFP,* *35S::BES1^S2A^-EYFP* and *35S::BES1^S2D^-EYFP* seedlings with or without 5 μM BL treatment.
