# Supplementary material for: CLE19 suppresses brassinosteroid signaling output via the BSL‐BIN2 module to maintain BES1 activity and pollen exine patterning in Arabidopsis
Source: J Integr Plant Biol. 2025 Aug 28;67(12):3216–30. doi: 10.1111/jipb.70024 (PMC12678687; doi:10.1111/jipb.70024)
Supplement: Supplementary file 5 — Table S1. Primers used in this study [file JIPB-67-3216-s004.pdf]

**Table S1. Primers used.**

| Primer Name | Primers(5`-3`) | Function |
|---|---|---|
| bri1-301 genotyping-LP | GGAAACCATTGGGAAGATCA | For genotyping of *bri1-301* |
| bri1-301 genotyping-RP | GCTGTTTCACCCATCCAA | For genotyping of *bri1-301* |
| det2-1 genotyping-LP | CTGAGAACAGGCGAGGTG | For genotyping of *det2-1* |
| det2-1 genotyping-RP | GTGGAGGAACAGCAAC | For genotyping of *det2-1* |
| bes1-D genomicDNA-LP-350bp | ACCCCAAACCATTGCCTACT | For genotyping of *bes1-D* |
| bes1-D genomicDNA-RP-350bp | TCTCCCCTTCCCATGGCTTA | For genotyping of *bes1-D* |
| NPTII-LP | TGGGCACAACAGACAATCGGCTGC | For genotyping of *CLE19-OX* |
| NPTII-RP | TGCGAATCGGGAGCGGCGATACCG | For genotyping of *CLE19-OX* |
| bil1-genotype1 | AGGTAACCTTATACATGGTAG | For genotyping of *bil1* |
| bil1-genotype2 | CTTACATGATTGAATTCTCTG | For genotyping of *bil1* |
| bil2-genotype1 | TCGTGCTCCAGAACTGATC | For genotyping of *bil2* |
| bil2-genotype2 | GCAAGGTCTATTGCTTCTG | For genotyping of *bil2* |
| LB-bil1bil2 | TTTCTCCATATTGACCATCATACTCATTG | For genotyping of *bil1* and *bil2* |
| bri1-5-genotypeF | GTGTACTTTCGATGGCGTTACCT | For genotyping of *bri1-5* |
| bri1-5-genotypeR | GGTTAAAGAAGCAGAGCA | For genotyping of *bri1-5* |
| BIN2-genotype1 | CTGTGATCTTGTCATTTAC | For genotyping of *bin2-1* |
| BIN2-genotype2 | TGAAGAAACCTCAAGACC | For genotyping of *bin2-1* |
| BIN2-genotype1 | CTGTGATCTTGTCATTTAC | For genotyping of *bin2-3* |
| BIN2-genotype2 | TGAAGAAACCTCAAGACC | For genotyping of *bin2-3* |
| LB-bin2-3 | CGTGTGCCAGGTGCCCACGGAATAGT | For genotyping of *bin2-3* |
| CS833298-LP | GAGATGGGAGGTTGTTTCCTC | For genotyping of *mee48-2* (CS833298) |
| CS833298-RP | AAATCCCAAATTCGAATTTGG | For genotyping of mee48-2(CS833298) |
| CS851736-LP | CACATGTCTGTTTCTTGCTGC | For genotyping of *acos5-4* (CS851736) |
| CS851736-RP | CACCGCTTCTACAAACGCTAC | For genotyping of *acos5-4* (CS851736) |
| CLE19-RT-FP | GATTCTTGCCTTCATTCATCAGTC | for real time-PCR of *CLE19* transcripts |
| CLE19-RT-RP | TTACCTGTTGTGGAGTGGATT | for real time-PCR of *CLE19* transcripts |
| DYT1-rt-FP | AACCAGTGAGGATGAGCCGTAGG | for real time-PCR of *DYT1* transcripts |
| DYT1-rt-RP | CGCAGAGCCATAAGCCGACA | for real time-PCR of *DYT1* transcripts |
| MYB35-rt-FP | GTCATAGAGCCATTGGCAGCAG | for real time-PCR of *MYB35* transcripts |
| MYB35-rt-FR | CGGGTCTATCCCCATTTTCATC | for real time-PCR of *MYB35* transcripts |
| AMS-rt-FP | TCACAAGGCTAATGGAGGCACT | for real time-PCR of *AMS* transcripts |
| AMS-rt-RP | TGTTCAGCTTGGACCATCTCG | for real time-PCR of *AMS* transcripts |
| ACOS5-rt-FP | ATGCGGAGATACCGACAA | for real time-PCR of *ACOS5* transcripts |
| ACOS5-rt-RP | GACCGATCATTTCAGACC | for real time-PCR of *ACOS5* transcripts |
| MEE48-rt-FP | AACATTGAGGATGGCTTGTG | for real time-PCR of *MEE48* transcripts |
| MEE48-rt-RP | GAGCCAATCCATTGAAGAAAC | for real time-PCR of *MEE48* transcripts |
| BES1-rt-FP | AACCACACTGTAAGTTAC | for real time-PCR of *BES1* transcripts |
| BES1-rt-RP | ACCGTTGATTTGAGTTAA | for real time-PCR of *BES1* transcripts |
| BRI1-rt-FP | CCGTGTACTTTCGATGGCGTTA | for real time-PCR of *BRI1* transcripts |
| BRI1-rt-RP | GAGAGACAGGAGAGACGAGGAC | for real time-PCR of *BRI1* transcripts |
| DWF4-rt-FP | AGGTGGGATTCTTGGGAAA T | for real time-PCR of *DWF4* transcripts |

| | | |
|---|---|---|
| DWF4-rt-RP | CTTTTTGGCCTCGTCTTGAG | for real time-PCR of *DWF4* transcripts |
| CPD-rt-FP | CGCTTTTCTCCTCCTCCTCT | for real time-PCR of *CPD* transcripts |
| CPD-rt-RP | CATGAAAACCGAACCGTACC | for real time-PCR of *CPD* transcripts |
| BR6OX2-rt-FP | TGTGGTTGGGATGATCTTG A | for real time-PCR of *BR6OX2* transcripts |
| BR6OX2-rt-RP | CTCCACTGCGGTAATTCGTT | for real time-PCR of *BR6OX2* transcripts |
| DET2-rt-FP | CCAATTGGAGTTGATTCTGCCC | for real time-PCR of *DET2* transcripts |
| DET2-rt-RP | ATTTCAGAAGGACGGCGGTT | for real time-PCR of *DET2* transcripts |
| ROT3-rt-FP | TGGATTGTATATAGTGAGATACGGGT | for real time-PCR of *ROT3* transcripts |
| ROT3-rt-RP | TCAAAAACCATGCAGGCAGAG | for real time-PCR of *ROT3* transcripts |
| DWF5-rt-FP | TCAGAATCTGAGGCTTTGGC | for real time-PCR of *DWF5* transcripts |
| DWF5-rt-RP | TGAGTAACAGAACCATCCTGATGA | for real time-PCR of *DWF5* transcripts |
| DWF1-rt-FP | TCAGGTACTCTCTCTACCTCTCT | for real time-PCR of *DWF1* transcripts |
| DWF1-rt-RP | AGTTGGTGAGCAAAGTTTGAAACT | for real time-PCR of *DWF1* transcripts |
| DWF7-rt-FP | CATTCATAAACTTGGTGACGTGGA | for real time-PCR of *DWF7* transcripts |
| DWF7-rt-RP | TGGCACACAGAGAGTTTGATGA | for real time-PCR of *DWF7* transcripts |
| CYP90D1-rt-FP | AACACAAACCCATTCGTGCG | for real time-PCR of *CYP90D1* transcripts |
| CYP90D1-rt-RP | CTTTGGTCCGTGACTCTGGG | for real time-PCR of *CYP90D1* transcripts |
| ACTIN-rt-FP | ATCGGTGGTTCCATTCTTGCTTC | for real time-PCR of *ACTIN* transcripts |
| ACTIN-rt-RP | TGGACCTGCCTCATCATACTCG | for real time-PCR of *ACTIN* transcripts |
| BSL1-insitu-FP | TCTTATTCTCTTCGGCGGCGCCACC | For insitu constructs of *BSL1* |
| BSL1-insitu-RP | ATGCCATCAGAACGAGCACTGCCAG | For insitu constructs of *BSL1* |
| BSL2-insitu-FP | CTGCGGTTGTTGGACAAGAGC | For insitu constructs of *BSL2* |
| BSL2-insitu-RP | CTGTGCCATCGTGGCCGTTG | For insitu constructs of *BSL2* |
| BSL3-insitu-FP | CAATGGTACCAGAGAATGATCAAGA | For insitu constructs of *BSL3* |
| BSL3-insitu-RP | CCACTTATTAGAAAGAACATCATAA | For insitu constructs of *BSL3* |
| BIN2-insitu-FP | TTCTCTCTCTATCGCCACAA | For insitu constructs of *BIN2* |
| BIN2-insitu-RP | CTTGCAAAACCTTCTTTATCG | For insitu constructs of *BIN2* |
| BES1-insitu-FP | AATTCCAGCGAAGAAGAAAGA | For insitu constructs of *BES1* |
| BES1-insitu-RP | TCGGGAATGAAGAAGAGGAA | For insitu constructs of *BES1* |
| BES1-pGWB441-FP | GGGGACAAGTTTGTACAAAAAAGCAGGCTTCATGACG TCTGACGGAGCAAC | For EYFP tag constructs of *BES1* |
| BES1-pGWB441-RP | GGGGACCACTTTGTACAAGAAAGCTGGGTCACTATGA GCTTTACCATT | For EYFP tag constructs of *BES1* |
| BES1-S219AS223A-pGWB441-FP | GGGGACAAGTTTGTACAAAAAAGCAGGCTTCATGACG TCTGACGGAGCAAC | For EYFP tag constructs of *BES1-S219AS223A* |
| BES1-S219AS223A-pGWB441-RP | GGGGACCACTTTGTACAAGAAAGCTGGGTCACTATGA GCTTTACCATT | For EYFP tag constructs of *BES1-S219AS223A* |
| BES1-S219DS223D-pGWB441-FP | GGGGACAAGTTTGTACAAAAAAGCAGGCTTCATGACG TCTGACGGAGCAAC | For EYFP tag constructs of *BES1-S219DS223D* |
| BES1-S219DS223D-pGWB441-RP | GGGGACCACTTTGTACAAGAAAGCTGGGTCACTATGA GCTTTACCATT | For EYFP tag constructs of *BES1-S219DS223D* |
| BES1-S219AS223A-FP | GTGGCTGCACCTGCCGCTCCTACTCATCATCGC | For site-directed mutagenesis of *BES1-S219AS223A* |
| BES1-S219AS223A-RP | AGGAGCGGCAGGTGCAGCCACCGCATAAAACGG | For site-directed mutagenesis of *BES1-S219AS223A* |
| BES1-S219DS223D-FP | GTGGATGCACCTGCCGATCCTACTCATCATCGC | For site-directed mutagenesis of *BES1-S219DS223D* |
| BES1-S219DS223D-RP | AGGATCGGCAGGTGCATCCACCGCATAAAACGG | For site-directed mutagenesis of *BES1-S219DS223D* |
